# A High-Resolution Inventory of Anthropogenic Methane Emissions in New York State

**DOI:** 10.1021/acs.est.5c07245

**Published:** 2025-08-07

**Authors:** Matthew L. Loman, Lee T. Murray, Eric M. Leibensperger, Joannes D. Maasakkers

**Affiliations:** † Department of Earth and Environmental Sciences, 6927 University of Rochester, Rochester, New York 14627, United States; ‡ Department of Physics and Astronomy, 6927 University of Rochester, Rochester, New York 14627, United States; § Department of Physics and Astronomy, 4102 Ithaca College, Ithaca, New York 14850, United States; ∥ 55944 SRON Netherlands Institute for Space Research, Leiden 2333 CA, Netherlands

**Keywords:** greenhouse gases, emissions inventory development, fossil fuels, natural gas, landfills, wastewater, fuel combustion

## Abstract

Anthropogenic sources of methane have become an important area of research in recent years, as subnational entities such as New York (NY) State mandate methane emission reductions to improve air quality and limit global warming. To facilitate modeling of regional methane emissions, we present an inventory of spatially disaggregated anthropogenic methane emissions in 2020 for NY at 100 m horizontal resolution and monthly temporal resolution. We distribute emissions for 82 source categories reported in statewide inventories from the NY State Department of Environmental Conservation (NYSDEC) and the New York State Energy Research and Development Authority (NYSERDA). This work compares favorably with existing gridded inventories including the New York City Urban Area inventory, but the NYSDEC and NYSERDA reports estimate total anthropogenic methane emissions that are 38 and 170% higher than the 2020 NY totals of the gridded Environmental Protection Agency inventory and version 8 of the Emissions Database for Global Atmospheric Research inventory, respectively, primarily due to emissions from fossil fuels and landfills. Although some major anthropogenic sources of methane remain uncertain, this work is foundational for the local-scale analyses and modeling that will be necessary for NY and other subnational entities to achieve their emission reduction targets.

## Graphical abstract

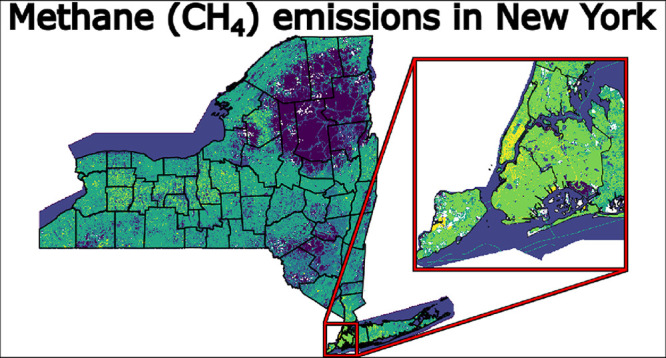

## 1 Introduction

Immediate large-scale reductions in greenhouse gas (GHG) emissions are necessary to limit climate change to an increase of 2 °C in global mean surface air temperature over preindustrial levels.^1^ New York (NY) State has implemented policies to reduce GHG emissions, including the Climate Leadership and Community Protection Act of 2019, commonly known as the “Climate Act,” which is one of the first statutory laws in the United States (US) to mandate GHG reductions. The Climate Act requires that NY reduce GHG emissions to 60% of 1990 levels by 2030.

Methane (CH_4_) is a powerful GHG and a significant precursor to the production of tropospheric ozone (O_3_), itself a greenhouse gas and a criteria pollutant regulated by the US Environmental Protection Agency (EPA). Although methane has a much shorter atmospheric lifetime (≈11 years) than carbon dioxide (CO_2_), it has greater affinity to absorb longwave radiation, giving it 82 ± 26 times the climate impact of an equivalent-mass emission of carbon dioxide (CO_2_) over a 20-year time frame, or 30 ± 11 times its impact over a 100-year time frame, as quantified by the Global Warming Potential (GWP) metric.^2^ Policymakers commonly use GWP-100, as in the EPA’s GHG inventories,^3^ but the Climate Act specifies that NY emissions be calculated using GWP-20, leading to increased importance of CH_4_ in budgeting.^4^ Using this metric, methane makes up 27% of all anthropogenic GHG emissions in NY in 2020.^4^ Implementing existing methane mitigation measures can reduce emissions by as much as 50%, slowing the rate of climate change by over 25% in the coming decades.^5^ Reducing methane emissions is, therefore, key to mitigating climate change and achieving the requirements of the Climate Act.

The Climate Act also requires the NY State Department of Environmental Conservation (NYSDEC) to produce annual Statewide GHG Emissions Reports (hereafter, “Statewide GHG Reports”).^4^ These reports provide emissions for individual source types aggregated at statewide totals, calculated using bottom-up methods.

Figure 1 compares the location of large landfills —major point sources of methane —in western NY with methane emissions from the updated Gridded EPA (GEPA2) inventory by Maasakkers et al.^6^ at 0.1° horizontal resolution. This illustrates the difficulties in local- and urban-scale applications that result from relatively coarse spatial resolutions, even when spatial and temporal information is available. For example, landfills near their counties’ borders can emit to a grid cell with most of its area in a different county, complicating aggregation of county-level (and thereby most policy-relevant) statistics from top-down optimizations. In this work, we address these challenges in NY by spatially disaggregating methane emissions from the NYSDEC Statewide GHG Reports at monthly temporal resolution and a horizontal resolution of 100 m, with a goal similar to that of Maasakkers et al.:^6^ preparing an a priori estimate of NY methane emissions to use in data assimilation. Our work is one of very few regional gridded inventories of greenhouse gas emissions available at such a fine spatial resolution and temporal resolution,^11–15^ and to our knowledge the first to be developed for any locale in North America.

**Figure 1:**
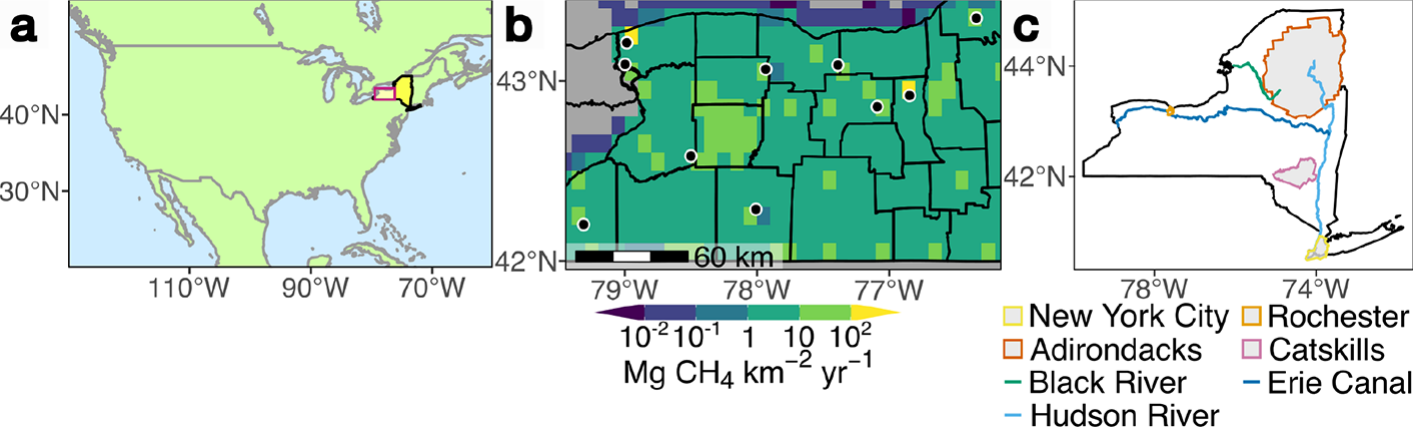
(a) Location of NY (yellow) in North America and location of subplot (b) in NY (red box). (b) Total 2018 anthropogenic methane emission flux from the updated gridded Environmental Protection Agency (EPA) inventory (GEPA2)^6^ compared with major landfill locations (black points) in western NY. Landfill coordinates are supplied by the EPA Greenhouse Gas Reporting Program (GHGRP)^7^ and confirmed with satellite imagery.^8^ (c) Map showing major geographical features of NY. Location data from the US Geological Survey,^9^ the NY Protected Areas Database,^10^ and satellite imagery.^8^

## 2 Methods

The NY State Energy Research and Development Authority (NYSERDA) has produced the *NY State Oil and Gas Sector Methane Emissions Inventory* ^16^ (OGSMEI) and the *Energy Sector Greenhouse Gas Emissions under the NY State Climate Act Report* ^17^ (ESGHGR) to supplement the NYSDEC Statewide GHG Reports (GHGR), which quantify methane emissions from the fossil fuel and energy sectors, respectively, in greater detail than the NYSDEC GHGR.

We spatially disaggregate the anthropogenic methane emissions reported in the 2022 NYSDEC GHGR, the 2022 NYSERDA OGSMEI, and the 2022 NYSERDA ESGHGR for 2020, the most recent year included in these reports, to a 100 m × 100 m resolution grid in the Universal Transverse Mercator (UTM) coordinate system Zone 18N (EPSG:26918). We choose this resolution and projection to facilitate data assimilation at spatial and temporal scales relevant for individual counties or metropolitan areas and because this is the official projection used for NY geographical information systems (GIS) data sets. Tables showing statewide 2020 methane emission totals for each source type in the NYSERDA and NYSDEC inventories, as reported at https://data.ny.gov (accessed 26 September 2024), can be found in Supporting Information Section S4. Since we prepare our gridded methane emission maps with inverse modeling in mind and since inverse model methodologies commonly are unable to redistribute emissions to areas where they do not already exist, we distribute low methane emissions (0.003% and 0.004% of total NY emissions) to two types of areas where we expect methane flux but are unsure of the magnitude. Details on these emissions are provided in Supporting Information Sections S1.1 and S1.2.3.

We adapt the methods of the gridded EPA inventories^6,18^ and the New York City Urban Area inventory (NY-UA)^19^ for much of our disaggregation work. We apply the same emission factors as the NYSDEC and the NYSERDA inventories wherever possible. We scale all of our emission totals to match those of the NYSDEC and NYSERDA inventories and generate monthly scaling factors when we expect seasonal variation in emissions. Additional detail on the methods described below is provided in Supporting Information Section S1. We discuss the limitations of the data we use for spatial distribution in Supporting Information Section S2.

### 2.1 Agriculture

The 2022 NYSDEC GHGR *Sectoral Report 3: Agriculture, Forestry, and Land Use* ^20^ reports statewide totals for agricultural emissions. Agricultural emissions of methane in the NYSDEC GHGR include enteric fermentation (160 Gg CH_4_ yr^–1^ in 2020) and manure management (76 Gg CH_4_ yr^–1^ in 2020).^20^ Both enteric fermentation and manure management emissions are separated by animal group in the NYSDEC GHGR. Methane emissions from rice cultivation and agricultural burning are not included in the NYSDEC GHGR so we do not include them in this work.

Due to federal privacy law, we are unable to indirectly publish livestock populations of individual farms or Concentrated Animal Feeding Operations (CAFOs), limiting the accuracy of our distribution of livestock emissions. Our approach focuses instead on identifying areas likely to contain livestock and estimating livestock population by area. Further discussion of the limitations of this approach is available in Supporting Information Section S2.1.

We first estimate the distribution of livestock populations using the United Nations Food and Agriculture Organization Gridded Livestock of the World maps,^21–26^ the 2019 National Land Cover Database,^27,28^ and county-level animal totals from the 2017 Census of Agriculture.^29^ We then distribute the NYSDEC GHGR’s enteric fermentation and manure emissions by estimated population for each animal group.

We calculate monthly scaling factors for our gridded maps of manure emissions using a temperature-dependency equation from Mangino et al.,^30^ following the methods of Maasakkers et al.^18^ (see Supporting Information Section S1.1 for details).

### 2.2 Fossil Fuel Systems

The NYSDEC GHGR includes energy sector emissions occurring outside NY that are associated with fossil fuels imported into NY (610 Gg CH_4_ yr^–1^ emitted outside NY in 2020).^4^ Here, we consider only fossil fuel system emissions occurring within NY as reported by the OGSMEI (170 Gg CH_4_ yr^–1^ in 2020).^16^ The OGSMEI includes methane emitted from oil and gas well activity, abandoned wells, gas compressor stations, gas storage, gas pipelines, and end-use in NY.

#### 2.2.1 Oil and Natural Gas Production

We use the Empire State Organized Geologic Information System database^31^ of oil and gas wells in NY for 2020 well and drilling data, including locations, completion dates, drill days, and production volume, following the methods of the OGSMEI.^16^ Table S2 shows the activity data we use for fugitive methane emissions from oil and gas production. We follow the methods described in the OGSMEI to calculate upstream emissions where possible.

#### 2.2.2 Natural Gas: Midstream Systems and Distribution

We use location and activity data purchased from the proprietary Rextag North America GIS data set for August 2022–2023 ^32^ for compressor stations, storage facilities, and gathering and transmission pipelines. Although there is publicly available location data for this infrastructure from the US Energy Information Administration (EIA),^33,34^ those data sets are not sufficiently accurate in space (see Supporting Information Section S1.2.2). Table S3 shows the activity data we use for fugitive methane emissions from oil and gas production.

Distribution pipelines are controlled by local gas utilities, and the corresponding location data is largely unavailable in the Rextag data product. We estimate total distribution emissions by material for each operator using the US Department of Transportation Pipeline and Hazardous Materials Safety Administration’s Gas Distribution Annual Data for 2020 ^35^ and emission factors based on the work of Lamb et al.^36^ reported in the OGSMEI. We then distribute to roads in natural-gas service areas for each gas-utility operator.^37,38^

#### 2.2.3 Natural Gas: End-Use Fugitives

End-use fugitive methane emissions include those from service meters, internal gas pipes, pilot lights, and appliances. Recent work suggests that natural-gas end-use fugitive emissions are severely underestimated in inventories, particularly in urban areas.^19,36,39–46^ Notably, because of the emission factors used for natural-gas end-use and combustion in the OGSMEI and the ESGHGR, and because it is not possible for measurements to isolate end-use fugitive emissions from combustion emissions, there is double counting of natural-gas combustion emissions in these inventories (see Supporting Information Section S1.2.3 for details). We still include both categories in this work to match the published NYSERDA emission totals, but advise caution to avoid double counting in future work.

We first distribute emissions from each natural-gas end-use category to counties using the proportion of state total carbon monoxide (CO) emissions from combustion of natural gas, reported by county and sector (residential, commercial/institutional, and industrial) in the EPA’s National Emissions Inventory,^47^ similarly to the methods used by Pitt et al.^19^ for stationary combustion emissions. We use CO emissions as a proxy for end-use fugitive emissions for greater spatial granularity compared to natural gas delivery data, which is only available from the EIA at the state level. We use the NY State Tax Parcel Centroid Points database^48^ (“Tax Parcels”) and 2020 US Census data^49^ to distribute end-use emissions within counties. Finally, we apply monthly scaling factors by sector based on consumption data from the US EIA.^50^

### 2.3 Solid Waste

The NYSDEC GHGR *Sectoral Report 4: Waste* reports totals for solid waste methane emissions (420 Gg CH_4_ yr^–1^ in 2020).^51^ However, 44% of inventoried methane emissions (190 Gg CH_4_ yr^–1^ in 2020) from solid waste management in the NYSDEC GHGR is from waste that originates in NY but is exported to landfills and solid waste combustion facilities outside NY,^51^ resulting in significant emission leakage. This work only distributes the 56% of solid waste methane emissions (230 Gg CH_4_ yr^–1^ in 2020) emitted within NY. Methane emissions from all waste in NY landfills, including waste imported from out-of-state, are calculated together the NYSDEC GHGR. As a result, we do not calculate separate emissions for imported waste. We note that recent studies indicate that bottom-up inventories tend to underestimate methane emissions from landfills.^45,46,52–55^

#### 2.3.1 Landfills

We distribute landfill emissions in NY using data from the Greenhouse Gas Reporting Program (GHGRP),^7^ the EPA Landfill Methane Outreach Program,^56^ annual reports from the NYSDEC,^57^ and the federal Facility Registry Service^58^ following methods similar to Maasakkers et al.^18^ All three databases contain coordinates for most landfills, which we use alongside open-source GIS software QGIS^59^ and satellite imagery^8^ to visually define geographical extents of all landfills in NY (see Supporting Information Section S1.3 for details).

We assign GHGRP-reported emissions to the 35 landfills that report to the GHGRP, which accounts for 47% of the NYSDEC GHGR total 2020 landfill emissions in NY. For the remaining landfills with waste data available from NYSDEC reports or the Landfill Methane Outreach Program, we estimate emissions using an equation from the 1990–2020 EPA GHGI, also used by the NYSDEC GHGR, that calculates emissions based on total waste-in-place.^51,56,57,60,61^ The remaining landfills, listed only in the Facility Registry Service, have only names and coordinates available. We calculate median fluxes for landfills with emission estimates and apply these to the landfills listed in the Facility Registry Service. Finally, we scale up the emissions estimates for landfills without GHGRP-reported emissions so that the state total matches the 2020 total NY emissions of landfill methane reported in the NYSDEC GHGR.

#### 2.3.2 Waste Combustion

We distribute emissions from waste combustion to solid waste management facilities containing the keyword “combustion” in the NYSDEC Solid Waste Management Facilities database.^62^ We distribute emissions reported in the NYSDEC GHGR^51^ proportionally by the amount of waste combusted at each facility from NYSDEC combustion reports.^63^

### 2.4 Wastewater

The NYSDEC GHGR *Sectoral Report 4: Waste* reports statewide totals for municipal wastewater emissions (31 Gg CH_4_ yr^–1^ in 2020).^51^ Although not included in the NYSDEC GHGR, Guisasola et al.^64^ and several subsequent studies^65–74^ have shown that non-negligible amounts of methane are emitted from sewers. We distribute some wastewater treatment plant emissions to their associated sewer networks to improve the accuracy of our gridded inventory without departing from the inventory totals calculated in the NYSDEC GHGR. For each wastewater category, we use resident and nonresident populations reported in the Clean Watersheds Needs Survey to estimate separate weekday (resident + nonresident) and weekend (resident only) emission totals.

#### 2.4.1 Treatment Plants

The NYSDEC GHGR only estimates wastewater treatment emissions for municipal plants, neglecting emissions from industrial wastewater treatment. We use the GHGRP-reported industrial wastewater emissions (0.26% of total NY wastewater treatment plant methane emissions reported by the NYSDEC) and coordinates, and distribute the remaining emissions to municipal wastewater treatment. We distribute municipal wastewater treatment emissions using facility coordinates and population served from the EPA’s Clean Watersheds Needs Survey.^75^ We estimate centralized wastewater emissions using the method described in the NYSDEC GHGR^51^ adapted from Bartram et al.^76^ This method calculates expected methane emissions per unit time as the product of population, expected biochemical oxygen demand (BOD) of waste produced per person per unit time, and methane conversion factors. For cities with populations over 50,000 and densely populated counties surrounding New York City (NYC), we distribute 90% of treatment plant emissions this way and reserve 10% for distribution to sewers as described in Section 2.4.2.

#### 2.4.2 Sewers

Comparative measurements suggest that sewers emit about 10% as much methane as their associated treatment plant.^77,78^ In the absence of any data collected in NY, we distribute 10% of treatment plant emissions to sewers of facilities in cities with populations over 50,000, as well as the highly populated counties near NYC (Nassau, Suffolk, and Westchester). We distribute 1.19 Gg CH_4_ yr^–1^ in this way. We distribute sewer emissions using data from public data on county Web sites or acquired via Freedom of Information Law requests submitted to county governments. We assume all sewer lines within a sewershed have equal emission rates per unit length, and when precise sewer locations are unavailable, we distribute to roads within the sewershed.^38^

#### 2.4.3 Septic Systems

We use the value of 10.7 g CH_4_ person^–1^ day^–1^ from Leverenz et al.^79^ as used in the NYSDEC GHGR alongside resident and nonresident populations from the Clean Watersheds Needs Survey to estimate septic system methane emissions. We adapt the methods of Pitt et al.^19^ to distribute these emissions to less-developed areas in the National Land Cover Database by their associated municipalities in the Clean Watershed Needs Survey.^27^ This process distributes 90% of the septic system emissions reported in the NYSDEC GHGR to areas covering the majority of NY; we distribute the remaining 10% of emissions to less-developed areas in the remainder of NY.

### 2.5 Other

#### 2.5.1 Iron and Steel

The NYSDEC GHGR *Sectoral Report 2: Industrial Processes and Product Use* reports statewide totals for industrial greenhouse gas emissions (130 kg CH_4_ yr^–1^ in 2020).^80^ The only methane source reported here is iron and steel production, with only one facility that was in operation in NY in 2020.^80^ We distribute the emissions reported in the NYSDEC GHGR to this facility using location data from the GHGRP.^7^

#### 2.5.2 Stationary Combustion

The ESGHGR reports statewide total methane emissions from fuel combustion by consumer type and fuel type.^17^ For each consumer type (electricity generation, nonresidential consumption, and residential consumption), we apply monthly scaling factors to emissions from stationary natural-gas combustion. We calculate these using NY 2020 monthly natural-gas delivery volumes to each consumer type, reported by the EIA, relative to the annual total for that consumer type in NY in 2020.^50^

We distribute electricity generation emissions (0.53 Gg CH_4_ yr^–1^ in 2020)^17^ using location and fuel type information from the EPA’s Power Sector Data Crosswalk.^81^ We use fuel input rate from the Power Sector Data Crosswalk, emission factors from the EPA Greenhouse Gas Inventory (GHGI),^60^ and GHGRP-reported emissions to estimate methane emissions.

We distribute residential and nonresidential stationary combustion emissions (commercial/institutional and industrial combustion: 4.4 Gg CH_4_ yr^–1^ in 2020; residential combustion: 12 Gg CH_4_ yr^–1^ in 2020)^17^ similarly to natural-gas end-use fugitive emissions described in Section 2.2.3, using residential and nonresidential stationary combustion CO estimates by county and fuel type from the EPA’s National Emissions Inventory and using Tax Parcels data^48^ for residential, commercial/institutional, and industrial locations (see Supporting Information Section S1.5.1).

#### 2.5.3 Mobile Combustion

The ESGHGR reports statewide totals for mobile combustion greenhouse gas emissions (4.4 Gg CH_4_ yr^–1^ in 2020).^17^ We use vehicle miles traveled data from the Federal Highway Administration 2020 Highway Statistics Series^82^ for NY to distribute on-road emissions to NY roads in the Topographically Integrated Geographic Encoding and Referencing system (TIGER) Roads database^38^ based on road type. We distribute aviation emissions to runway areas provided by the Federal Aviation Administration^83^ by the number of flights from the associated airport reported by the Bureau of Transportation Statistics^84,85^ for each month. We distribute rail emissions to railroads in NY in the TIGER Rails database.^86^ Methane emissions from fuel consumed in the operation of gas pipeline distribution networks are also included in the ESGHGR.^17^ We distribute these emissions equally to all compressor stations using the same locations as in Section 2.2.2. We assume nonroad military transportation primarily occurs within military-owned properties and distribute these emissions by area to defense infrastructure listed in the NY State Federal Properties GIS database.^87^ We assume nonroad commercial and industrial transportation primarily occurs on commercial and industrial properties and distribute these emissions to those properties as listed in the Tax Parcels database.^48^ We distribute all other off-road mobile emissions using National Land Cover Database^27^ land types based on the off-road emission category (see Supporting Information Section S1.5.2).

## 3 Results and Discussion

Figure 2 shows our Gridded New York State (GNYS) inventory of 2020 NY total anthropogenic methane flux and 2020 NY methane flux subtotals for each of the five main source categories in Section 2 remapped to 0.05° × 0.05° resolution for plotting. Figures S5–S10 show subregions of the 2020 NY anthropogenic methane flux gridded maps of total emissions and the five main source categories for the NYC and Rochester, NY metropolitan regions at their native 100 m horizontal resolution. Figure 1C shows major geographical features of NY that are referenced in this section when describing the distribution of methane emissions.

**Figure 2:**
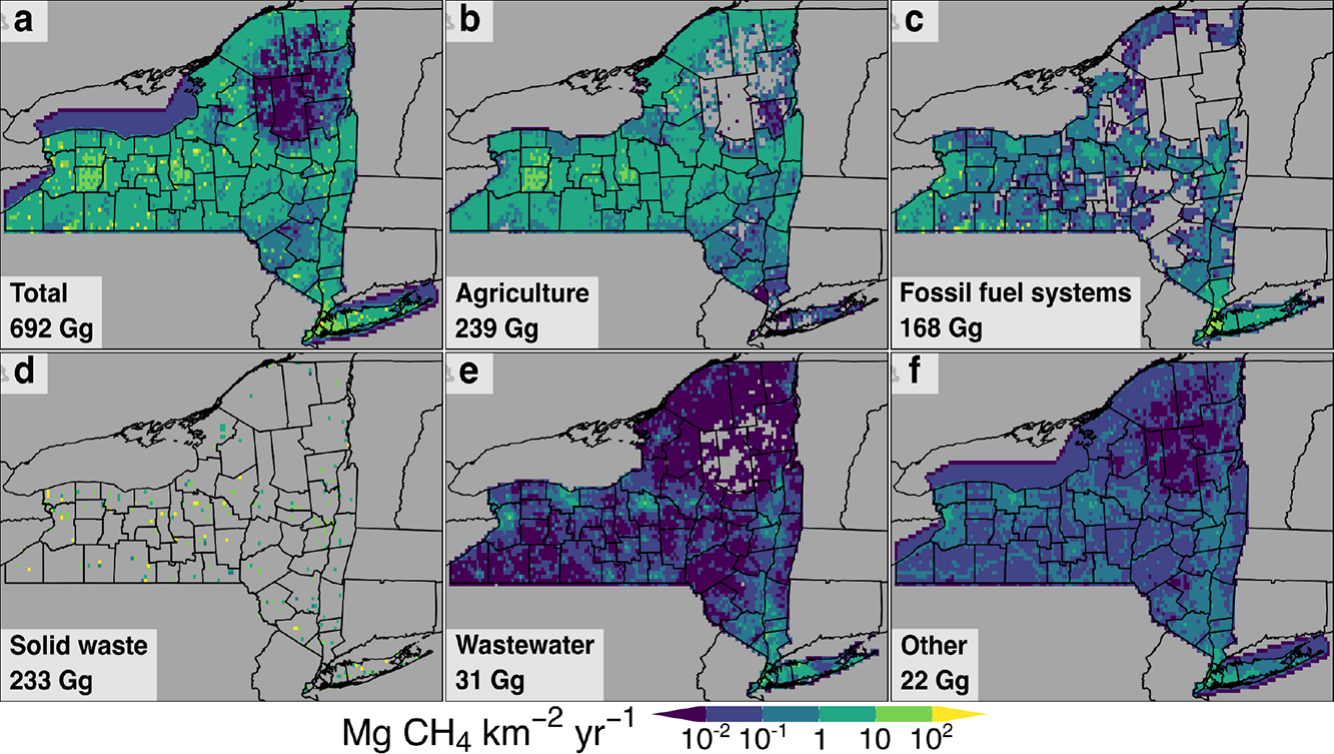
Spatially disaggregated annual 2020 anthropogenic methane emission fluxes in NY in the Gridded New York State (GNYS) inventory. (a) Total emissions; (b–f) emissions from the five source categories described in Section 2. Data shown here is remapped from native 100 m resolution to 0.05° × 0.05° resolution for plotting using tools from the Geospatial Data Abstraction Library (GDAL)^88^ and Climate Data Operators (CDO).^89^ NY emission total and subtotals for 2020 are shown in each subplot. Source category subtotals do not sum to total due to rounding errors. Black lines show state/provincial and NY county borders.

Figure 2B shows the methane flux from agriculture. These emissions are spread throughout NY with the exception of the Adirondack Mountains in the northeast, the Catskill Mountains in the southeast, and urban areas. Over 80% of 2020 NY agricultural methane emissions come from dairy cattle. Hotspots of agricultural emissions in rural western and central NY and the Black River valley correspond with counties containing the highest cattle populations in the state.

Figure 2C shows methane flux from fossil fuel systems, which is concentrated in population centers (NYC, the Hudson Valley, and along the Erie Canal corridor) due to leaks from gas-distribution infrastructure and end-use fugitive emissions, which together make up over one-third of emissions in this category. Emissions from gas distribution and end-use are excluded from areas without gas-utility service, leading to the patchy distribution of emissions of Figure 2C. Fossil fuel production leads to methane emission hotspots in southwestern NY, mainly from low-producing conventional gas wells. Compressor stations occur as point sources of high emissions along transmission pipelines, and constitute nearly 40% of fossil fuel infrastructure emissions. Offshore gas pipelines within NY borders lead to elevated emissions in NY Harbor and Long Island Sound (Figure S5).

Figure 2D shows methane flux from landfills, the most concentrated sources of anthropogenic methane in this inventory. Landfills are scattered across the state with the exception of mountainous and urban areas. The western half of the Erie Canal corridor hosts the five largest active landfills in NY. In this work, we estimate that over one-quarter of the landfills in NY each emitted over 1 Gg yr^–1^ of methane in 2020. Emissions of GHG from waste combustion, by contrast, are primarily CO_2_ and therefore account for less than 0.5% of 2020 methane emissions from solid waste.^51^

Figure 2E shows wastewater–methane flux. Wastewater treatment plants make up 48% of total 2020 NY wastewater–methane emissions which appear as point sources statewide, with greater density in more heavily populated areas such as the Hudson Valley. Sewer methane emissions (5%) which we distribute throughout large cities and densely populated regions in NY, visible as areas of greater emissions along the Erie Canal corridor and in the NYC metropolitan area. The remainder of wastewater emissions come from septic systems which appear as a background of diffuse emissions.

Figure 2F shows methane flux from other anthropogenic sources. Industry is responsible for a negligible proportion of 2020 methane emissions from the “Other” category; all emissions visible in Figure 2F are from combustion, 79% from stationary sources and 21% from mobile sources. Stationary combustion emissions are most visible in the same large urban areas to which we allocate sewer emissions, and primarily come from residential and commercial sources (71 and 21%, respectively). The distribution of combustion emissions through the remainder of the state primarily reflects on-road transportation (73% of mobile combustion emissions). Low methane emission fluxes surrounding Long Island, over the Great Lakes, and over other large lakes result from mobile fuel combustion for boating. The boundaries of emissions over water correspond with the boundaries of NY.

Statewide emission totals for all 82 individual source types are available at https://data.ny.gov/ (accessed 26 September 2024) and are reproduced in tables in Supporting Information Section S4.

### 3.1 Uncertainty

The New York inventories on which our Gridded New York State (GNYS) inventory is based do not include estimates of uncertainty in the inventory totals. We instead use the relative error for different emissions categories reported for 2020 in the 1990–2020 EPA GHGI,^60^ since the methods of the New York inventories are largely similar.^4,16,17^ We apply the following equation from Maasakkers et al.^6^ to estimate total uncertainty σ­(τ) as a function of resolution for each methane source.

$$\sigma \left(\tau \right)=\sigma_{\tau }\left(\tau \right)+\sigma_{N}=\sigma_{R}\times \exp\left[-k_{\tau }\left(\tau -\tau_{0}\right)\right]+\sigma_{N}$$ 1

where τ is grid cell resolution in degrees, σ_τ_ is the resolution-dependent uncertainty of the source emissions due to spatial disaggregation methods, σ_*R*_ is σ_τ_ at τ = τ_0_ = 0.1° in the GEPA2 inventory, *k*_τ_ is the error decay coefficient which captures the decrease in error from spatial disaggregation as grid box size increases, and σ_*N*_ is the uncertainty of the total NY emissions of the source, equal to the reported 95% confidence intervals as a percentage of total source emissions from the EPA GHGI for the inventory year.^90^ To estimate σ_*R*_ and *k*_τ_, Maasakkers et al.^6^ compared their updated Gridded EPA (GEPA2) methane inventory to a finer-resolution gridded inventory developed for a subset of their domain that matched well with observations. We are not aware of another gridded inventory for NY that fits these criteria. Therefore, we use the σ_*R*_ and *k*_τ_ values reported by Maasakkers et al.^6^ to broadly estimate resolution-dependent uncertainty. More detail, including a table of the values used, is provided in Supporting Information Section S3. As a result of the method used, the relative uncertainties calculated for this work at its native resolution are very conservative, with a weighted average uncertainty across all emission categories of 93%. Further work, such as uncertainty estimation in NY inventories and top-down evaluation of this gridded inventory, is necessary to establish a more reliable evaluation of uncertainty in the GNYS inventory.

### 3.2 Comparisons

Table 1 shows details of three independently developed gridded inventories of bottom-up methane emissions and our GNYS inventory. In this section, we compare our work with these three inventories and discuss the drivers of their differences. We compare our work with the New York City Urban Area inventory (NY-UA)^19^ separately from our comparisons with the updated Gridded EPA Inventory (GEPA2)^6^ and the Emissions Database for Global Atmospheric Research version 8.0 (EDGARv8)^91^ since the NY-UA inventory does not cover the entirety of NY, while the GEPA2 and EDGARv8 products do, and because it is available at a much higher spatial resolution.

**Table 1:** Details of Three Independently Developed Gridded Inventories and This Work (GNYS)^a^

inventory	spatial resolution	temporal resolution	years available	domain	citation
GEPA2	0.1°	monthly	2012–2020^b^	continental US	^6^
EDGARv8	0.1°	annual	1970–2022	global	^91^
NY-UA	0.02°	annual	2019	NYC MSA^c^	^19^
GNYS	100 m^d^	monthly	2020	NY	this work

a We compare the results from this work with those of the updated Gridded EPA Inventory (GEPA2)^6^ and the Emissions Database for Global Atmospheric Research version 8.0 (EDGARv8)^91^ in Section 3.2.1, and with the results of the New York City Urban Area inventory (NY-UA)^19^ in Section 3.2.2.

b Full gridded inventory available 2012–2018. Years 2019 and 2020 are available through the ”Express Extension” which scales 2018 values to match totals for those years.

c NYC Metropolitan Statistical Area and surroundings, 39.2°–42.0°N, 72.1°–75.7°W.

d Approximately 0.0012° × 0.0009°.

#### 3.2.1 The Gridded EPA Inventory (GEPA2) and the Emissions Database for Global Atmospheric Research Version 8.0 (EDGARv8)

Table 2 shows NY methane emissions reported by the updated Gridded EPA Inventory (GEPA2)^6^ for 2020 via the “Express Extension” that is consistent with EPA GHGI for 2020,^60^ the European Commission’s Emissions Database for Global Atmospheric Research version 8.0 (EDGARv8)^91^ for 2020, and the NYSDEC and NYSERDA inventories for 2020.^4,16,17^ Methane emission totals for NY in our GNYS inventory match the 2020 totals of the NYSDEC and NYSERDA inventories by design. For NY emission totals for this work (GNYS), relative uncertainties are equal to the emission-weighted EPA GHGI^60^ confidence intervals (σ_*N*_ in eq 1), as the totals in Table 2 are not dependent on our spatial allocation of emissions. To estimate relative uncertainties for NY total emissions in the GEPA2 inventory, we apply eq 1 to each emission source, estimating NY-aggregate σ_τ_ by calculating the resolution in degrees of a single grid cell with the area and latitude of NY. We then calculate emission-weighted averages for the categories in Table 2. The resolution dependence of uncertainty in NY totals in the GEPA2 inventory but not in the GNYS inventory leads to higher values for relative uncertainty for the GEPA2 inventory despite our use of the same methodology.

**Table 2:** Annual 2020 Total NY Methane Emissions by Category from the Gridded EPA Inventory (GEPA2) Express Extension for 2020,^6^ the Emissions Database for Global Atmospheric Research Version 8.0 (EDGARv8),^91^ and This Work (“GNYS”)^a^

category	IPCC	methane emissions in Gg yr^–1^				
		GEPA2		EDGARv8	GNYS^b^	
agriculture	4	230_–4.2_^+13^	16%	43_–2.4_^+3.6^	240	16%
fossil fuel systems	1B	120_–18_^+30^	48%^c^	52_–5.4_^+8.6^	170	26%
solid waste	6A, 6C	110_–15_^+4.0^	26%	100_–9.2_^+43^	230	23%
wastewater	6B	20_–5.5_^+4.6^	26%	32_–5.1_^+5.5^	31	29%
other^d^	1A, 2C	21_–2.7_^+2.7^	72%	26_–5.3_^+3.8^	22	66%
**total** ^e^		**500** _–44_^+56^	29%	**260** _–34_^+58^	690	23%

a For the GEPA2 and the EDGARv8 inventories, values are calculated assuming constant flux within grid cells that cross the NY border and are therefore proportional to cell area within NY. Reported upper and lower bounds assume all or none of the emissions in those cells occur in NY, respectively. Percentages indicate 1 σ uncertainty for the GEPA2 and GNYS inventories. See text for details.

b Emission totals for each category match those of the NYSDEC^4^ and NYSERDA^16,17^ inventories.

c Uncertainty parameters recommended in Maasakkers et al.^6^ for stationary combustion applied to end-use fugitives, as in Supporting Information Section S3.

d Fuel combustion and industry (Section 2.5).

e Values do not sum to totals due to rounding errors.

The NY inventories report higher methane emissions from fossil fuel systems and solid waste than both the GEPA2 and the EDGARv8 inventories, while agriculture, wastewater, fuel combustion, and industrial methane emission totals from the NY inventories all agree with at least one of the two independently developed gridded anthropogenic methane inventories. This results in large differences in total anthropogenic methane emissions in NY, although this difference does not reach statistical significance between the GNYS and GEPA2 inventories. The lower emission total of the EDGARv8 inventory cannot be evaluated for statistical significance here due to the lack of numerical uncertainty reporting by sector in the EDGARv8 inventory.

Solid waste emissions are the only category for which the difference between the GEPA2 and GNYS inventories is statistically significant (*p* = 0.02). The NYSDEC GHGR, the EPA GHGI, and the EDGARv8 inventory all use the same equation from the IPCC^76^ for estimating landfill emissions; however, the EPA GHGI also allows the use of a different method for landfills that have gas capture systems, which estimates methane emissions based on the gas collected.^92^ Recent satellite inversions suggest that emissions calculated using this gas capture method are are biased low.^45,54,55^ The EPA GHGI uses emissions from these equations only for landfills reporting to the GHGRP and applies a scaling factor of 1.09 to account for all other landfill emissions, while the NYSDEC solid waste inventory applies the IPCC equation to all waste produced in the state rather than scaling the GHGRP-reported emissions. The results suggest either that the methods of the EPA GHGI fail to capture a large proportion of landfill emissions in the United States, as has been indicated by several recent observational studies,^45,46,52–55^ or that, relative to the US average, a larger proportion of waste in NY is in smaller landfills that do not report to the GHGRP. For many countries, including the United States, the EDGARv8 inventory uses data reported to the United Nations Framework Convention on Climate Change (UNFCCC). With the existing documentation of the EDGARv8 inventory, it is difficult to assess whether the agreement in NY solid waste emissions between the EDGARv8 and GEPA2 inventories is due to the use of the same UNFCCC-reported activity data or other factors.^91^

The EDGARv8 inventory estimates much lower agricultural methane emissions for NY than the GEPA2 or NYSDEC GHGR inventories. All three inventories use IPCC “Tier 2” methods for cattle and “Tier 1” methods for other livestock.^20,91,92^ However, livestock methane emission totals over the entire continental United States for the GEPA2 and EDGARv8 inventories compare favorably: 9.37 Tg CH_4_ yr^–1^ (75% enteric fermentation, 25% manure) in 2020 in the GEPA2 Express Extension and 9.20 Tg CH_4_ yr^–1^ (74% enteric fermentation, 26% manure) in 2020 in the EDGARv8 inventory. In the EDGARv8 inventory, 89% of NY livestock emissions are from enteric fermentation and 11% from manure, compared to 68 and 32% in this work, and 63 and 37% in the GEPA2 inventory. Although the proportion of enteric fermentation emissions is much higher in the EDGARv8 inventory in NY than in the continental United States as a whole, total EDGARv8 methane emissions from both enteric fermentation and manure are an order of magnitude lower in NY in the EDGARv8 inventory compared to the GEPA2 inventory and this work. The EDGARv8 inventory cites FAOSTATS for livestock distribution data, but does not specify whether this means the Gridded Livestock of the World maps, which agree with the USDA-reported population totals that support the GEPA2 inventory, the NYSDEC GHGR, and this work (see Supporting Information Section S1.1).

Methane emissions from fossil fuel systems in NY are very different in all three inventories, although this difference does not reach statistical significance between the GNYS and GEPA2 inventories. As mentioned in Section 2.2.3, the underestimation of methane emissions from fossil fuel systems in the EPA GHGI has been established by several recent studies,^19,36,39,40,42–46^ so the larger value reported by the NYSERDA inventory in Table 2 is unsurprising. Despite their differences in NY, the GEPA2 and EDGARv8 inventories allocate similar fossil fuel systems emissions to the entire continental United States: 9.8 Tg CH_4_ yr^–1^ in 2020 in the GEPA2 inventory and 10 Tg CH_4_ yr^–1^ in 2020 in the EDGARv8 inventory. The proportions of emissions from oil, gas, and coal production both nationwide and in NY are also similar in the two inventories. In NY, which has no coal production in 2020, these emissions are 6.7% oil and 93% gas in the GEPA2 inventory, and 8.6% oil and 91% gas in the EDGARv8 inventory. The variety of methods to spatially distribute these emissions, the use of some proprietary data in both works, and differences in the categories in which emissions are reported make it difficult to explain the large difference in total NY fossil fuel systems emissions between these inventories. The major driver of the difference in these emissions between our work and the GEPA2 gridded inventory is compressor station emissions, which may be overestimated the OGSMEI, as indicated in submitted work by Ravikumar et al.^93^ We provide a more detailed comparison of our work with the GEPA2 inventory in Supporting Information Section S4.1 thanks to its greater transparency and more numerous subcategories than the EDGARv8 inventory. End-use fugitive emissions of natural gas in NY (“post-meter” emissions in the EPA GHGI) are very similar in the OGSMEI (19 Gg CH_4_ yr^–1^ in 2020) and the GEPA2 inventory Express Extension (19 Gg CH_4_ yr^–1^ in 2020). The EPA GHGI included postmeter emissions for the first time for the 1990–2020 GHGI,^60^ but recent work indicates that these emissions remain severely underestimated in the EPA GHGI and the GEPA2 Express Extension,^45^ suggesting that the same is true of these emissions in the OGSMEI and this work.

Figure S1 shows the difference between gridded total anthropogenic methane emission fluxes from this work, remapped to 0.1° horizontal resolution, and those from the GEPA2 and the EDGARv8 inventories in NY. The pattern of methane emissions statewide matches well between the three inventories. The GEPA2 and NY inventories are similar in both distribution and magnitude due in part to overlap in the data sets used for spatial disaggregation. The largest spatial differences between this work and the GEPA2 and the EDGARv8 inventories represent landfills, gas compressor stations, and gas wells. These are the largest contributors to the two categories with the greatest differences in total emissions between the NY inventories and the GEPA2 and the EDGARv8 inventories, shown in Table 2. The EDGARv8 inventory uses population to distribute emissions from compressor stations, whereas this work and the GEPA2 inventory treat these as point sources.^6,16,91,94^ Larger emissions in this work relative to the EDGARv8 inventory in the NYC metropolitan area are primarily a result of natural-gas distribution infrastructure emissions, which are significant in this area.

Due to an apparent error in the EDGARv8 solid waste inventory, in which landfill emissions are assigned to the wrong grid cell, this work shows much lower emissions relative to the EDGARv8 inventory in some grid cells in Figure S1D. Figure S4 illustrates this error.

Figure 3 shows the monthly variability of methane emissions in NY in this work and the GEPA2 and EDGARv8 inventories. Figure S11 compares monthly variability for individual emission categories. We use 2018 data for monthly emissions in the GEPA2 inventory since some monthly variability in the GEPA2 inventory is not available for the 2020 Express Extension.^6^ Solid waste and wastewater emissions are aggregated here due to the way monthly data is reported in the EDGARv8 inventory. For the GEPA2 and GNYS inventories, seasonal changes in agricultural emissions due to the temperature dependence of methane emissions from manure management dominate month-to-month changes in total methane with the same pattern due to use of the same equation, while combustion emissions dominate the monthly variability of methane emissions in the EDGARv8 inventory. Monthly variability in agricultural emissions is slightly greater in the GEPA2 inventory than in this work because we only apply monthly scaling to manure managed as a liquid, while the GEPA2 inventory estimates monthly variability for all manure management emissions. The EDGARv8 inventory applies some temperature-dependency to seasonal manure management emissions, but with an amplitude of less than 10%.^95,96^ Fossil fuel systems emissions in the GEPA2 inventory vary within the year based on production volumes, which have an increasing trend in 2018. By contrast, this work estimates monthly variability in fossil fuel systems for end-use fugitive emissions only based on gas consumption volume by sector (electricity generation, commercial, industrial, and residential). The EDGARv8 inventory does not include monthly variability in fossil fuel systems emissions.^95,96^ For combustion emissions, we again use gas consumption data by sector to apply monthly scaling to gas combustion emissions, and use reported flight data at NY airports for monthly aviation emissions. The GEPA2 inventory generates monthly fuel combustion scaling factors only for electricity generation using monthly data from the EPA’s Acid Rain Program.^6,97^ The EDGARv8 inventory provides more detailed seasonal scaling for fuel combustion, using reported consumption data for electricity generation, and heating degree days for residential and commercial combustion, and assuming a strong seasonal cycle for mobile combustion from agricultural machinery.^95,96^ The EDGARv8 inventory documentation indicates that seasonal cycles in road usage are applied for Europe, but it is unclear whether these patterns are extrapolated globally.^95,96^

**Figure 3:**
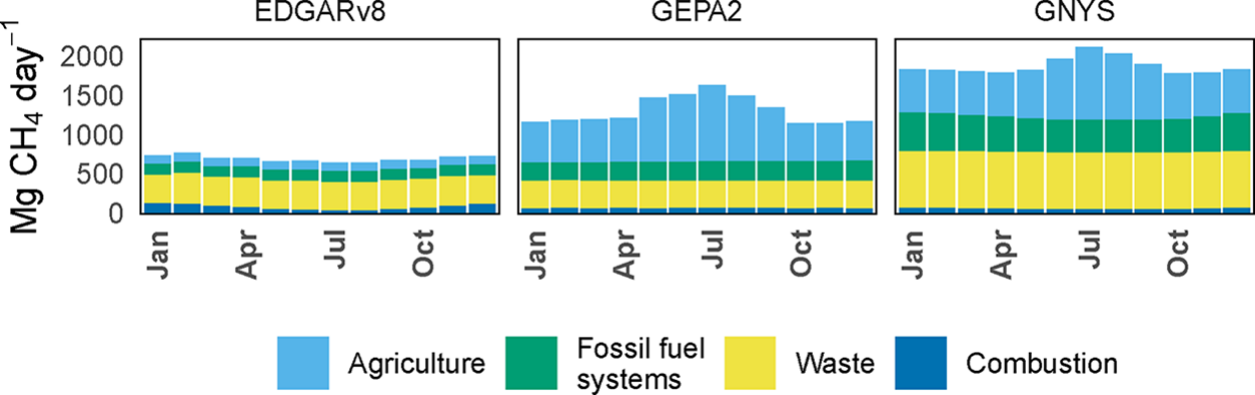
Stacked-bar plots of monthly methane emissions in NY in this work (GNYS) and the EDGARv8 and GEPA2 inventories. For the GEPA2 inventory, 2018 data is shown since monthly resolution is not complete for the 2020 data.

Supporting Information Section S4.1 contains further comparison of emission totals and spatial patterns in the this work, the GEPA2 Express Extension, and the EDGARv8 inventory, including statistical comparisons.

#### 3.2.2 The New York City Urban Area Inventory (NY-UA)

The New York City Urban Area (NY-UA) inventory is an independently developed, spatially resolved methane inventory gridded at 0.02° × 0.02° for a rectangular area containing the NYC metropolitan area and its immediate surroundings (39.2°–42.0°N, 75.7°–72.1°W).^19^ The NY-UA inventory consists of 144 versions that use different combinations of sources for calculating and distributing emissions. The authors selected four (referred to in Pitt et al.^19^ as HRA, HRB, HRC, and HRD) for detailed analysis based on their correlation with measured methane fluxes and their spatial granularity of calculations.^19^ Supporting Information Section S4.2 contains further details on the differences between these four versions of the NY-UA inventory. Here, we compare with the NY-UA inventory version HRB because of its greater correlation with observations relative to version HRD and its use of smaller areas for calculating category totals before disaggregation relative to versions HRA and HRC;^19^ however, this work compares similarly to all versions of the NY-UA inventory.

Table 3 shows anthropogenic methane emissions from version HRB of the New York City Urban Area (NY-UA) inventory of Pitt et al.^19^ and this work (GNYS) in the overlap of their domains. We calculate different emission totals for the GNYS inventory in Table 3 than in parts above because here we can only compare using the part of this work that is within the domain of the NY-UA inventory. Similarly, the NY-UA inventory totals in Table 3 differ from those reported by Pitt et al.^19^ because we can only compare our work with the portion of the NY-UA inventory within the state of NY. Figure S2 in Supporting Information Section S4.2 shows the location of this area. Supporting Information Section S4.2 also contains tables matching Table 3 for the other three main versions of the NY-UA inventory. We do not compare our work with emissions in the “Other” category in the NY-UA inventory because those emissions were taken directly from the Gridded EPA Inventory for 2012.^18^ As in the comparisons with the GEPA2 and EDGARv8 inventories above, methane emissions from landfills and natural-gas infrastructure dominate the differences between the gridded inventories.

**Table 3:** Anthropogenic Methane Emissions by Category from Version HRB of the New York City Urban Area Inventory^19^ (NY-UA) and This Work (GNYS) in the Overlap of Their Domains^a^

category	methane emissions in Gg yr^–1^	
	NY-UA (2019)	GNYS (2020)
landfills	17_–.21_^+0.0054^	46
natural-gas distribution	28_–1.3_^+0.71^	32
natural-gas postmeter^b^	31_–1.4_^+0.36^	6.8
natural-gas transmission	3.0_–0.67_^+0.17^	11
stationary combustion–fossil fuels	4.2_–0.28_^+0.58^	2.7
stationary combustion–wood	4.3_–0.18_^+0.071^	3.9
wastewater	22_–3.0_^+0.70^	20
**total** ^c^	**110**_–1.6_^+8.7^	**130**

a “Total” includes only the source categories shown here; NY-UA emission categories for which gridded emissions are taken directly from the GEPA2 inventory are excluded. For the NY-UA inventory, values are calculated assuming constant flux within grid cells that cross the NY border and are therefore proportional to cell area within NY borders. Upper and lower bounds assume all or none of the emissions in those cells occur in NY, respectively.

b Residential only.

c Source category subtotals do not sum to total due to rounding errors.

Inversions performed by Pitt et al.^19^ using their NY-UA gridded inventory as a prior estimate resulted in posterior total emission rates for the NYC metropolitan area that were 1.7–1.9 times those of the prior. The similarity in inventory totals for the area considered in Table 3 indicates that this work also underestimates total methane emissions in the NYC metropolitan area. Pitt et al.^19^ found a mean posterior emission fraction for fossil methane of 0.69, compared to an average of 0.6 for their prior estimates (we calculate 0.59 for the portion of NY-UA version HRB within NY) and 0.44 for GNYS within the area considered in Table 3. Notably, if 90–120 Gg CH_4_ yr^–1^ (70–90% of the total for that area, corresponding to the posterior increase in emissions found by Pitt et al.^19^) of fossil methane is added to the GNYS totals for the area considered in Table 3, the resulting fraction of fossil methane for the area is 0.67–0.70, closely matching the posterior results of Pitt et al.^19^ This indicates that nonfossil methane emissions in the NYC area in this work are comparable to those of the posterior results of Pitt et al.,^19^ and suggests that total methane emissions in the NYC area in this work are underestimated primarily due to the well-documented underestimation of fossil methane emissions in national and regional inventories.^36,39–46^ For the remainder of this section, we diagnose the major differences between this work and the NY-UA inventory with the understanding that total emissions, and fossil methane emissions in particular, are likely underestimated in both inventories. Supporting Information Section S4.2 contains additional details on these comparisons.

Figure S2 shows the difference between anthropogenic methane emission fluxes in NY-UA version HRB and this work. Both inventories assign large fluxes to NYC (bottom center of each subplot of Figure S2) and smaller fluxes to its surroundings, but the dominance of fossil emissions in the NY-UA inventory and that of landfill emissions in this work lead to more diffuse fluxes in the NY-UA inventory and more high-emission hotspots in this work.

The NY-UA inventory includes only landfills that report to the GHGRP^7^ or the Landfill Methane Outreach Program,^56^ estimating emissions from landfills found only in the Landfill Methane Outreach Program by applying a constant emission rate of approximately 11 Mg CH_4_ yr^–1^ to each landfill, calculated using the remaining landfill emissions inventoried by the 1990–2019 EPA GHGI^98^ after subtracting the GHGRP emissions.^19^ Six landfills in this area report to the GHGRP and therefore have similar total emissions in both gridded inventories, averaging 1362 Mg CH_4_ yr^–1^ per landfill in this work, but we allocate an average of 1141 Mg CH_4_ yr^–1^ to the landfills of the Landfill Methane Outreach Program in this area, and we also allocate emissions to 29 additional landfills in the inventories’ shared domain. The NY-UA inventory uses 0.1° gridded methane emissions from the GEPA^18^ inventory for industrial landfills, which are visible in Figure S2 as large rectangles. For municipal landfills, the NY-UA inventory uses point data while we distribute emissions to the entire surface area. For landfills appearing in both inventories, this leads to large differences surrounding the cell containing the coordinates associated with the landfill (Figure S2).

Although the OGSMEI and the NY-UA inventory both cite Fischer et al.^99^ for residential postmeter emissions, the OGSMEI uses a per-housing-unit emission factor from Fischer et al.^99^ while the NY-UA inventory applies their residential postmeter emission factor of 0.5% of gas usage to consumption data from the EIA^100^ and distributes in HRB using residential CO_2_ emissions from the Anthropogenic Carbon Emission System (ACES) v2 ^101^ maps. As an intermediate step in creating the NY-UA inventory, Pitt et al. estimated total NY emissions for residential postmeter emissions; the proportion of this estimate they allocated to this part of NY matches the proportion in this work (0.66).^102^ This indicates that the large difference shown in Table 3 is driven by uncertainty in total natural-gas postmeter emissions, underscoring recent findings that natural-gas postmeter fugitive emissions are under-reported.^39–41^ We also include fugitive emissions from appliances in this category, for which the OGSMEI uses emission factors from Merrin and Francisco^103^ and the EIA’s Residential Energy Consumption Survey.^104^

Compressor stations make up 94% of gas transmission emissions in the OGSMEI. The OGSMEI uses a constant emission rate per compressor station, while the NY-UA inventory scales GHGRP-reported emissions so that their mean matches a default emission rate. The NY-UA inventory calculates their default rate using total US compressor station count and total compressor station emissions from the 1990–2019 EPA GHGI,^98^ which itself uses the same emission factors from Zimmerle et al.^105^ as the OGSMEI. However, as a result of incomplete Rextag data for compressor station locations (discussed in Supporting Information Section S4.1), our distribution of compressor station emissions within NY is limited to those with known locations. Greater compressor station emissions in this work also lead to scattered cells with very high differences in emissions between the two inventories (Figure S2).

Methane emissions from combustion, natural-gas distribution, and wastewater contribute little to the differences in emissions, partly due to similar disaggregation methods. Supporting Information Section S4.2 contains comparisons of this work and the NY-UA inventory for these categories, as well as statistical comparisons for all emission categories.

### 3.3 Implications

We present the Gridded New York State (GNYS) inventory, a spatially and temporally disaggregated inventory of anthropogenic methane emissions across New York State at 100 m horizontal resolution and monthly temporal resolution for all sectors, with weekday/weekend resolution for select sectors. By design, our inventory’s 82 source categories and their annual emission totals are consistent with those reported in the inventories developed for 2020 by the state: the NYSDEC *Statewide Greenhouse Gas Emissions Reports*, the NYSERDA *NY State Oil and Gas Sector Methane Emissions Inventory*, and the NYSERDA *Energy Sector Greenhouse Gas Emissions under the NY State Climate Act Report*. We find that our methods of distributing methane emissions yield results that are consistent with independently developed regional, national, and global gridded methane inventories. The largest differences between inventories occur in methane emissions from landfills and fossil fuel systems, particularly natural-gas infrastructure. This aligns with previous findings that these sources have high uncertainty and tend to be underestimated in inventories such as the EPA GHGI.^19,36,39–46,52–55,106^ Despite the substantially larger methane emissions from fossil fuel systems in the NYSERDA inventory (and, therefore, this work) relative to the EDGARv8 and GEPA2 gridded inventories, our comparison with the NY-UA gridded inventory and the inversion results of Pitt et al.^19^ suggests that our work still underestimates these emissions in the NYC urban area.

Our high-resolution estimates of methane emissions can support policymakers with the planning and action necessary to achieve the goals of New York’s Climate Act by directing attention toward areas where emission mitigation would have the greatest impact. Our work also facilitates the use of atmospheric measurements and satellite retrievals within inverse modeling frameworks to further constrain methane emissions in New York. As such, we plan to continue this work by improving the end product based on our comparisons with other inventories, including the use of updated compressor station counts and emission factors from Ravikumar et al.^93^ We also plan to assimilate methane observations from within and around the state to provide optimized emission inventory estimates to stakeholders.

Although the NY state government provides a large amount of GIS data, we conducted the majority of this work using public databases available throughout the United States. The exception is the use of one proprietary input data set, which we found crucial for constraining in-state spatial patterns from the energy sector; nevertheless, lack of spatial data on natural gas distribution systems remains a major challenge. Our methodology may be applied to other municipalities to construct similar high-resolution inventories of anthropogenic methane to assist emission reduction efforts and atmospheric modeling. We found it challenging to distribute methane emissions from livestock and fossil fuel usage and downstream infrastructure due to limited available location data. Furthermore, although waste data from individual landfills was accessible via permitting records, we found a lack of publicly available data summarizing waste input for multiple landfills. We encourage government entities to publish additional data to facilitate further research.

The NYSDEC *Statewide Greenhouse Gas Emissions Reports*, the NYSERDA *NY State Oil and Gas Sector Methane Emissions Inventory*, and the NYSERDA *Energy Sector Greenhouse Gas Emissions under the NY State Climate Act Report* all lack numerical characterization of error. As such, we recommend that the category-specific uncertainty estimation methods described by Maasakkers et al.^6^ based on error reported in the EPA GHGI^60^ be applied to this study for applications where error characterization is necessary, as demonstrated in Section 3.1. We recommend that future statewide inventories report confidence intervals for each source category to improve their utility in top-down scientific applications.

The New York inventories used here also lack quantification of some known anthropogenic sources of methane. Emissions not estimated in New York inventories to date include those from sewers, composting, agricultural burning, and the use of manure as fertilizer. To facilitate data assimilation, we have attempted to account for most of these emissions by redistributing some methane emissions to areas where we expect these sources to occur. Since this method effectively reduces methane emissions from some source known in greater detail, we recommend that sources of methane missing from existing New York inventories be included in subsequent inventories without perturbing other sources (e.g., adding, rather than redistributing, a percentage of methane emissions to account for emissions from an under-studied source). We also recommend that these sources be studied in greater depth.

Finally, we recommend that the New York state government continue to support the development of spatially disaggregated methane emission inventories, either as a process incorporated into the development of the annual emission inventories cited here, or via funding of scientific research. This support would allow for updates to this inventory to match emission totals for other years and, therefore, improved capacity for atmospheric modeling and emissions analysis at a variety of spatial scales, thereby contributing to New York’s achievement of its emission reduction goals.

## Supplementary Material



## Data Availability

Gridded annual emission maps at native resolution for all 82 source types, as well as monthly and weekday/weekend scaling factors for applicable source types, are available at https://doi.org/10.5281/zenodo.16761163.

## Notes

Additional details on methodology, data limitations, and uncertainty calculations, and comparisons with the GEPA2, EDGARv8, and NY-UA inventories; tables containing the methane emission totals for NY from the NYSDEC and NYSERDA reports as used in this work; figure demonstrating the need for use of private sources for oil and gas location data; figure demonstrating the inaccuracy of landfill emission locations in the EDGARv8 inventory; figures of gridded emissions at native resolution for selected locations; and figures showing side-by-side comparisons and differences of gridded emissions in this work and the GEPA2, EDGARv8, and NY-UA inventories (PDF)
