# Supplementary material for: A High-Resolution Inventory of Anthropogenic Methane Emissions in New York State
Source: Environ Sci Technol. 2025 Aug 7;59(32):16933–46. doi: 10.1021/acs.est.5c07245 (PMC12368985; doi:10.1021/acs.est.5c07245)
Supplement: Supplementary file 1 [file es5c07245_si_001.pdf]

Supporting information for “A high-resolution inventory of anthropogenic methane emissions in New York State”

Matthew L. Loman,[*,†] Lee T. Murray,[†,‡] Eric M. Leibensperger,[¶] and Joannes D. Maasakkers[§]

†*Department of Earth and Environmental Sciences, University of Rochester, Rochester, NY 14627 USA*

‡*Department of Physics and Astronomy, University of Rochester, Rochester, NY 14627 USA*

¶*Department of Physics and Astronomy, Ithaca College, Ithaca, NY 14850 USA*

§*SRON Netherlands Institute for Space Research, Leiden 2333 CA NLD*

E-mail: mloman@ur.rochester.edu

Summary: 57 pages, 28 figures, 25 tables.

Contents

S1 Detailed Methods **S4**

S1.1 Agriculture S4

S1.2 Fossil Fuel Systems S8

S1.2.1 Oil and Natural Gas Production S8

S1.2.2 Natural Gas: Midstream Systems and Distribution S9

S1.2.3 Natural Gas: End-Use Fugitives S12

S1.3 Solid Waste S15

S1.4 Wastewater S17

S1.4.1 Treatment Plants S17

S1.4.2 Sewers S18

S1.4.3 Septic Systems S18

S1.5 Other S19

S1.5.1 Stationary Combustion S19

S1.5.2 Mobile Combustion S21

S2 Data Limitations **S22**

S2.1 Agriculture S22

S2.2 Fossil Fuel Systems S23

S2.3 Solid waste S23

S2.4 Wastewater S24

S2.5 Other S24

S3 Uncertainty **S25**

S4 Detailed Comparisons **S26**

S4.1 The Gridded EPA Inventory (GEPA2) and the Emissions Database for Global Atmospheric Research version 8.0 (EDGARv8) S26

S4.2 The New York City Urban Area Inventory (NY-UA) S32

S5 Source category tables **S41**

S6 Additional Figures **S46**

S6.1 GNYS source categories at native resolution S47

S6.2 GEPA2 Comparisons S51

S6.3 EDGARv8 Comparisons S53

S6.4 NY-UA Comparisons S55

References **S58**

S1 Detailed Methods

Here we provide additional details on the methods described in Section 2 of the main text. Limitations of these methods are discussed in Section S2.

We choose to project our gridded inventory in the Universal Transverse Mercator (UTM) coordinate system Zone 18N (EPSG:26918) because the UTM system uses a conformal projection that preserves angles and shapes across smaller regions. Its use of narrow zones with separate projection parameters also means that distance is nearly conserved; over the area of New York, local distortion of distance is less than 0.2 %.[1]

S1.1 Agriculture

The updated gridded EPA inventory (GEPA2) inventory allocates 4.8×10^4 kg CH_4 yr^{-1} to agricultural burning within New York in 2018, with 47 % of these emissions coming from a group of four western counties. This suggests that although agricultural burning is a small source of methane in New York (4.8×10^4 kg CH_4 yr^{-1} is 0.02 % of the magnitude of statewide total agricultural emissions reported by the NYSDEC), it may be relevant on local scales. However, despite reporting methane emissions for source categories with smaller state totals in its inventories, the NYSDEC Statewide GHG Report does not estimate methane emissions from agricultural burning. As a result, these emissions are not included in this work.

The 2017 US Census of Agriculture indicates no rice cultivation in New York except for one operation each in the New York City Boroughs of Queens and Staten Island (Queens and Richmond Counties, respectively).[2] The most recent EPA Greenhouse Gas Inventory (GHGI) reports negligible (less than 0.05 million metric tons of CO_2 equivalent) emissions from rice cultivation in New York in 2020,[3] so we likewise assume this source is insignificant. Data from the updated gridded EPA inventory (GEPA2) suggests that agricultural burning may be a non-negligible source of methane in some areas of New

York;[4] see Section S2.1 for more details.

Animal groups emitting less than 10^4 metric tons CO_2 equivalent (CO_2e) of methane in a given category do not have totals published in the NYSDEC Statewide GHG Report. We distribute enteric fermentation emissions from dairy cattle, beef cattle, horses, sheep, goats, and captive deer and elk, and manure management emissions from dairy cattle, beef cattle, swine, chickens, and horses.

Livestock population estimates from Gridded Livestock of the World gridded at $0.083^\circ \times 0.083^\circ$ summed over New York counties are well-correlated with the county totals from the Census of Agriculture (see Table S1). The National Land Cover Database is a gridded map of land cover in the contiguous United States based on Landsat satellite imagery at its native 30 m horizontal resolution coupled with various supporting datasets.[5,6] The Census of Agriculture provides county totals of animal populations for a wide variety of livestock, including categorizing cattle into dairy cows, beef cows, steers, and calves.

We use National Land Cover Database[5] raster values labeled as "pasture/hay" (index 81) and "grassland/herbaceous" (index 71) to select land for possible livestock use. However, this does not capture animal feeding operations (discussed further in Section S2.1). We remove from consideration all cells within protected areas using the New York Protected Areas Database,[7] except for those marked as agricultural or ranch easements. We equally distribute livestock populations estimated in the Gridded Livestock of the World cells to "grass/pasture" cells and then scale county totals to match totals from the Census of Agriculture. Scaling county totals allows us to use the finer spatial resolution of the Gridded Livestock of the World data without reproducing its bias in horse and pig populations shown in Table S1. Without separate layers for dairy and beef cattle in the Gridded Livestock of the World maps, we assume the ratio of dairy cows (female cattle) to beef cows equals the ratio of dairy cattle to beef cattle for lack of additional information. For farmed deer and elk, which are not included in Gridded Livestock of the World, we distribute county population totals from the Census of Agriculture equally among all

"grass/pasture" cells in the counties with farmed deer and elk.

We distribute all enteric fermentation emissions by animal population estimated as described above. All manure management emissions are also allocated in this way for all animal groups except chickens, for which we use this method for 99 % of emissions and distribute 1 % equally to all cells identified in the National Land Cover Database as containing crops to account for methane emissions from manure applied as fertilizer. Meta-analysis by Shakoor *et al.*[8] suggests that poultry manure significantly increases methane emissions when used as fertilizer, while Shakoor *et al.*[8] and several other studies not included in their meta-analysis[9–11] find that fertilizer use of manure from cattle and swine does not significantly impact methane emissions. However, one study shows a small but significant decrease in soil uptake of methane from the use of cattle manure as fertilizer and notes (but does not quantify) peaks in methane flux after its application.[12] In the absence of published relationships to quantify the impact of fertilizer application of manure on methane flux, we choose 1 % (2.8×10^4 kg CH_4 yr^{-1}) as an arbitrary small proportion of methane emissions from chicken manure and distribute equally to all cells in the National Land Cover Database with raster values labeled as "cropland" (index 82). We distribute the remaining 99 % (2.8 Gg CH_4 yr^{-1}) of methane emissions from chicken manure by estimated animal population described in the main text and above. For a gridded map that distributes 100 % of chicken manure emissions by estimated animal population, a factor of 100/99 may be applied to the gridded map of chicken manure emissions.

We apply monthly scaling factors to account for the temperature dependency of methane emissions from systems that manage livestock manure as a liquid. We first determine the proportion of methane emissions that result from these manure management using the proportion of animals with a given type of manure management system from Table A-3A2.1 of Wightman and Woodbury[13] and volatile solid production rates by animal group and methane emission factors for volatile solids by management system and

animal type from the IPCC's 2019 inventory guidelines.[14] For dairy cattle, we also use animal weights from the IPCC inventory guidelines. We apply monthly scaling factors only to methane emissions from "liquid," "deep pit," and "underboard" manure management systems for dairy cattle, other cattle, swine, and chickens. We use the following equation from Mangino *et al.*[15] to calculate temperature-dependent proportion of solids f available for conversion to methane in anaerobic animal waste lagoons:

$$f = \exp\left[\frac{E_a(T_a - T_0)}{RT_0T_a}\right] \tag{1}$$

where E_a is the activation energy constant (64 kJ mol^{-1}), R is the ideal gas constant (8.314 J K^{-1} mol^{-1}), T_0 is the base temperature at which $f = 1$ (303 K), and T_a is the ambient air temperature. We apply a lower limit temperature of 280.65 K to account for heat generated by biological activity as described in Annex 3B of the 2020 EPA GHGI.[16] We use monthly surface temperature gridded at 0.5°×0.625° horizontal resolution from the National Aeronautics and Space Administration (NASA)'s Modern-Era Retrospective analysis for Research and Applications, Version 2 (MERRA-2) reanalysis product[17] to calculate monthly mean values for f at 0.5°×0.625° horizontal resolution. We scale the results such that the time-weighted mean value is equal to 1 for each 0.5°×0.625° cell. Finally, we use tools from the Geospatial Data Abstraction Library (GDAL)[18] to remap the scaling factor rasters to match the projection and resolution of our gridded emission maps.

Table S1: Comparison of New York county livestock population totals in the 2017 Census of Agriculture[2] and the United Nations Food and Agriculture Organization Gridded Livestock of the World maps.[19–24] Percent difference indicates Gridded Livestock of the World county totals of animal population relative to Census of Agriculture reported values.

	Cattle	Chickens	Goats	Horses	Pigs	Sheep
R^2	0.99	0.99	0.95	0.98	0.60	0.99
% difference [a]	-5.4	-3.2	-4.1	250	38	-5.5

S1.2 Fossil Fuel Systems

S1.2.1 Oil and Natural Gas Production

Table S2: Activity data used to distribute fugitive methane emissions from oil and gas production.

Emission source	Activity data used
Well pads	Volume of oil or gas produced
Completions	Count
Drill rigs	Drill days
Drilling fugitives	Drill days
Mud degassing	Drill days
Abandoned wells	Count

High-volume unconventional production of oil and gas (also known as “hydraulic fracturing” or “fracking”) has been banned in New York since 2015,[25] and the Oil and Gas Methane Inventory indicates zero methane emissions from hydraulic fracturing from 1990 to 2020. The Oil and Gas Methane Inventory indicates zero methane emissions from mud degassing or completions from gas wells. All emissions in these categories are, therefore, distributed to oil wells only.

For any well reporting more than 50 drilling days, we set drilling days to 22 to adjust for outliers, consistent with the treatment in the New York State Energy Research and Development Authority (NYSERDA) Oil and Gas Methane Inventory.[26] The Oil and Gas Methane Inventory groups oil and gas well-pad emissions into high-producing and low-producing wells with different emission factors based on production volume. Using the criteria reported in the Oil and Gas Methane Inventory, we find no high-producing oil wells in the Empire State Organized Geologic Information System despite the inclusion of emissions for high-producing oil wells in the Oil and Gas Methane Inventory. This may be a result of differences in calculations of production rate since the Oil and Gas Methane Inventory’s cutoff values are based on daily production while the Empire State Organized Geologic Information System reports only the annual production total and number of active months for each well. However, the Oil and Gas Methane Inventory does not specify how it

calculates daily production values from the Empire State Organized Geologic Information System data. We calculate daily production by multiplying production months by the average number of days in a month (30.5 for 2020) and dividing annual production by this value. High-producing oil wells account for 1 % of all oil well-pad emissions in the Oil and Gas Methane Inventory. In the absence of location data for the high-producing oil wells mentioned in the Oil and Gas Methane Inventory, we distribute total methane emissions from all oil well pads by production using production and location data in the Empire State Organized Geologic Information System, rather than separately distributing emissions from high- and low-producing oil wells.

Commercial conventional extraction of fossil fuels in New York has been ongoing since the 1820s.[27] As a result of the long history of well drilling in New York, locating abandoned oil and gas wells in the state is an ongoing challenge, and the New York State Department of Environmental Conservation (NYSDEC) estimates that there remain as many as 30,000 wells in New York with no records in their registers,[28,29] limiting the accuracy of both the Oil and Gas Methane Inventory emission totals and their spatial distribution in this work. Currently, abandoned oil and gas wells account for less than 1 % of total oil and gas production emissions in the NYSERDA Oil and Gas Methane Inventory.[26]

S1.2.2 Natural Gas: Midstream Systems and Distribution

Table S3: Activity data used to distribute fugitive methane emissions from midstream systems for oil and gas

**Emission source**	**Activity data used**
Compressor stations	Count
Liquefied natural gas (LNG) storage	Facility count
Gathering & processing	
Gas	Compressor station count
Oil	Volume produced
Pipelines	
Gathering	Pipeline length
Transmission	Pipeline length
Distribution	Road length

In the interest of matching the methods of the Oil and Gas Methane Inventory, we do not use the emissions reported by the EPA's Greenhouse Gas Reporting Program (GHGRP) for compressor stations; instead, we apply the emission factors reported in the Oil and Gas Methane Inventory. Emissions from liquefied natural-gas (LNG) terminals and gas processing are not included in the Oil and Gas Methane Inventory since there are none of these facilities in New York.[26]

We assume that the majority of distribution mains and services are located under or near roads and, therefore, allocate emissions from distribution pipelines by road length within each service area using the US Census Bureau's Topographically Integrated Geographic Encoding and Referencing system (TIGER) roads database,[30] filtering out large expressways, alleys, and unpaved roads which we assume are unlikely to have pipelines running underneath. The TIGER Roads database includes attributes called "MAF/TIGER Feature Class Codes" that indicate road type.[30] To remove large highways and unpaved roads from this dataset, we exclude features with the following MAF/TIGER Feature Class Codes: S1100, S1500, S1630, S1640, S1710, S1720, and S1730. For New York City, features with MAF/TIGER Feature Class Code S1200 were also excluded. We use NY State Department of Public Service data to apply distribution pipeline emissions by gas utility operator to their respective natural-gas service areas.[30,31]

The US Department of Transportation Pipeline and Hazardous Materials Safety Administration's Gas Distribution Annual Data for 2020[32] reports distribution pipeline mains by material and length, as well as services by material and count for each operator. We use the average service length reported by each operator to determine the total service pipeline length by material, and then apply the emission factors from the Oil and Gas Methane Inventory to estimate total main and service pipeline emissions. The Oil and Gas Methane Inventory emission factors do not differentiate between coated and uncoated steel pipelines, so we use the same emission factors for both cathodically protected and unprotected steel pipelines. We use the emission factor for cast iron pipelines for recondi-

tioned iron and ductile iron pipelines, and apply the emission factor for protected steel pipelines to pipelines with material listed as "other." Using company names in the two datasets, we assign the resulting emission totals for operators listed in the data from the Pipeline and Hazardous Materials Safety Administration to the service territories listed in the New York State Gas Utility Service Territories shapefile.[31] We assume locations based on the following available information for two utilities that appear in the data from the Pipeline and Hazardous Materials Safety Administration but not the Service Territories shapefile: NEA Cross of NY, Inc. operates in Erie County (Buffalo) according to the New York State Department of Public Service,[33] so we add its emissions to the National Fuel Gas territory, which covers nearly all of Erie County. We distribute emissions from the Village of Hamilton Municipal Gas Utility to the area of the Village of Hamilton using a shapefile from New York State GIS Clearinghouse.[34] We assign emissions from the Pipeline and Hazardous Material Safety Administration pipelines listed under "Keyspan" and "Niagara Mohawk Power Corp" to National Grid for the relevant areas since these companies were acquired by National Grid. We assign emissions from pipelines listed under "Village of Hamilton Municipal Gas Utility" in the Pipeline and Hazardous Material Safety Administration data to the Village of Hamilton using the New York State Civil Boundaries shapefile.[34] N.E.A. Cross of New York, Inc. has information for about 70 miles of pipelines in the Pipeline and Hazardous Material Safety Administration dataset but no corresponding gas service territory.[31,32] N.E.A. Cross of New York, Inc. is included in the New York State Department of Public Service website as an active gas utility in Erie County, so we add the emissions from these pipelines to the total for National Fuel Gas Distribution, whose service territory according to the Department of Public Service covers nearly all of Erie County.[31] We also add the emissions calculated for the less than 20 miles of pipeline belonging to National Fuel Gas Supply Corp. to the National Fuel Gas Distribution territory as they belong to the same parent company and National Fuel Gas Supply Corp. has no associated service territory in the Pipeline and Hazardous Material

Safety Administration dataset.

S1.2.3 Natural Gas: End-Use Fugitives

In the NYSERDA Oil and Gas Methane Inventory, methane emissions from appliances include exhaust emissions from appliance startup, shutdown, and continuous operation.[26,35] Methane emissions from residential, commercial, and industrial gas use are also estimated by the NYSERDA Energy Sector GHG Report as stationary combustion emissions using emission factors borrowed from the 1990-2021 EPA GHGI,[36] which itself uses the Tier 1 emission factors for stationary combustion from the 2006 Intergovernmental Panel on Climate Change (IPCC) Emission Factors Database.[37] The 2006 IPCC Tier 1 emission factors do not account for sub-optimal combustion conditions. The Oil and Gas Methane Inventory uses appliance-specific emission factors from Merrin and Francisco,[35] and although their continuous operation measurements were taken as close as possible to maximum load conditions, the wide range in emissions per unit gas consumed suggests that optimal combustion conditions were not always reached. The use of both the IPCC combustion emission factors in the Energy Sector GHG Report together with the end-use fugitive emission factors from Merrin and Francisco[35] in the Oil and Gas Methane Inventory means that there is double counting of natural-gas combustion emissions.

The Oil and Gas Methane Inventory includes emissions from service meters from all buildings, but post-meter emissions are limited to residential buildings, restaurants, and hospitals. The Oil and Gas Methane Inventory does not estimate separate gas meter fugitive emissions for commercial and industrial sectors. For nonresidential buildings, we distribute 99 % of emissions to restaurants and hospitals using the emission factors from the Oil and Gas Methane Inventory, and for the remaining 1 %, we distribute evenly to all other nonresidential buildings to include some fugitive emissions beyond the meter in those structures. For a gridded map that distributes 100 % of nonresidential post-meter emissions to restaurants and hospitals, a factor of 100/99 may be applied to the gridded

map of restaurant and hospital post-meter emissions.

We categorize properties as either residential or nonresidential based on metadata in the New York State Tax Parcels database[38] to distribute these emissions. We use the New York State Gas Utility Service Territories data[31] to exclude areas outside gas service territories. We distribute emissions to buildings either listed as being serviced by a gas utility or with utilities unlisted because some counties choose not to provide this data, notably those in New York City. We reproduce the methods of the Oil and Gas Methane Inventory to distribute all end-use emissions, except for appliances, due to data availability. We assume one gas meter per nonresidential building in the absence of data to indicate meters per structure and apply the emission factors listed in the Oil and Gas Methane Inventory. For residential buildings, we estimate the housing unit count using building codes and distribute residential end-use emissions by housing unit using the emission factors reported in the Oil and Gas Methane Inventory for residential buildings and service meters, assuming one gas meter per housing unit. For residential emissions from natural-gas appliances, we also distribute emissions by housing unit in the absence of detailed spatial data on gas appliance usage.

Table S4 and **Table S5** show the categories used to group properties for the purpose of distributing natural gas end use emissions. Documentation describing the classification codes used in the "PROP_CLASS" and "BLDG_STYLE' attributes is available from the New York State Department of Taxation and Finance,[39] the New York City Department of City Planning,[40] and the New York City Department of Finance.[41] Links to these sources are also included in the New York State Tax Parcels Data Dictionary.[38]

For each building listed in the New York State Tax Parcels database[38] that is classified as residential, we distribute gas meters by estimated number of housing units, assigning one meter to each unit. We allocate X units to properties with property code description "X-family home" / "X-unit apartment" where X is a single number, not a range. We allocate Y units to each building with property code description containing a range of families or

units (*e.g.*, 5-6) where Y is the midpoint of the given range. We distribute remaining gas meters by estimating housing units in those multi-unit properties with unspecified number of units. We construct estimates by subtracting the sum of estimated housing units from housing units reported for each county in the 2020 US Census[42] to get number of housing units in multi-unit properties and distributing to multi-unit properties based on assessed property value ("TOTAL_AV") for those that have listed property value. We assign the county mean of estimated housing units in multi-unit properties for multi-unit properties with unlisted assessed property value or less than 1 unit in a building based on the above calculation. In the case of multi-unit properties with no nonzero property values listed for the entire county, we use the mean number of units for all buildings of that property class in New York excluding New York City for counties outside New York City, or the mean number of units for all buildings of that property class in the five counties (boroughs) of New York City.

Table S4: Attributes and values used to categorize properties as residential, commercial, and industrial for natural gas end use emissions in New York with the exception of New York City. The "#" character denotes all possible values.

**Building type**	**Attribute**	**Included**	**Excluded**
Residential	PROP_CLASS	2##, 410, 411, 481, 482	242
Commercial	PROP_CLASS	4##, 6##, 51#, 54#	44#, 410, 411, 437, 438, 439, 473, 474, 475, 513, 651, 653
Restaurants	PROP_CLASS	42#, 481, 482	
Hospitals	PROP_CLASS	641	
Industrial	PROP_CLASS	71#	

We calculate monthly scaling factors for a given consumer group (residential, commercial, and combined commercial and industrial) by dividing the Energy Information Administration reported monthly total New York 2020 natural gas delivery (Tables 14 through 16 in EIA Natural Gas Monthly[43]) by the mean of monthly natural gas delivery for 2020, weighted by the number of days in each month. This yields scaling factors with a time-weighted mean of 1 so that the time-weighted mean of the resulting gridded maps is identical to the 2020 annual mean gridded map.

Table S5: Attributes and associated values we use to categorize properties as residential, commercial, and industrial for natural gas end use emissions in New York City only. The "#" character denotes all possible values.

Building type	Attributes	Included	Excluded
Residential	BLDG_STYLE	A#, B#, C#, D#, L#, R#, S#, H6, H7	R0, R5, RA, RB, RG, RH, RK, RP, RS, RT, RW
Commercial	PROP_CLASS & BLDG_STYLE	PROP_CLASS: 04, 05, 07, 08	BLDG_STYLE: T2, U1
Restaurants	BLDG_STYLE	C7, D6, D7, D9, K5, K6, L1, L8, R7, R8, R9	
Hospitals	BLDG_STYLE	I1	
Industrial	BLDG_STYLE	06	

S1.3 Solid Waste

The NYSDEC Statewide GHG Report acknowledges that composting of organic waste is a source of methane but does not estimate the associated emissions, so they are excluded from this work as a result.

The Facility Registry Service includes many types of facilities; we filtered for landfills and classified them as municipal or industrial using keywords in the name and facility description fields. We use the following keywords to identify landfills: "landfill", "dump". We use the following keywords to identify industrial landfills: "company", "construction", "paper", "tire", "mining", "foundry", "industrial", "manufacturing", "mfg", "enterprise".

The procedure we use for visually identifying landfill extent is as follows. In most landfills, particularly those that are active or recently capped, the area in which waste is stored has characteristic features of disturbance that are visible from satellite imagery. These include an absence of trees and shrubs, smooth flanks with linear ridges, leachate ponds with angular borders, and access roads that encircle the landfill or form concentric loops moving up its slope. Using these features, we define landfill extent as the area in which waste is stored, most typically meaning the area within the outermost access road plus any leachate ponds when they are present and identifiable. If multiple distinct such areas are present in a single site, we use multiple polygons to define the extent. When these

characteristics are indistinguishable in older inactive landfills and dumps, we attempt to reproduce the above using visible patterns of disturbance and traces of access roads. When no disturbed area can be identified, as for many of the locations listed in the Facility Registry Service database, we exclude the location from our dataset. None of the landfills listed in the EPA's Greenhouse Gas Reporting Program (GHGRP) or the Landfill Methane Outreach Program were subject to exclusion.

Waste data is available from the NYSDEC reports as annual waste added for 27 large landfills starting in 2017;[44] however, most of these also have emissions estimated in the GHGRP. For landfills with annual waste data available from the NYSDEC and no GHGRP estimate, we estimate emissions with equation A-68 from Annex 3 of the 1990-2020 EPA GHGI, the same equation used by the NYSDEC Statewide GHG Report and the GHGRP.[45–47] This equation requires annual rainfall data to determine the decay constant, for which we used a mean of the National Oceanic and Atmospheric Administration (NOAA) annual precipitation values for 1991-2020[48] gridded at $0.25^\circ \times 0.25^\circ$ horizontal resolution.

The Landfill Methane Outreach Program reports total waste-in-place estimates from a single year, typically the year of closure.[49] Most landfills included in the Landfill Methane Outreach Program also report emissions to the GHGRP, so we use data from landfills with both waste-in-place estimates and methane emission estimates from the GHGRP to create an emission factor which we use to estimate emissions at the landfills that have only waste-in-place estimates available. For landfills with a closure year listed before 2020, we include an exponential decay factor by using the same rainfall-dependent decay constant recommended by the EPA GHGI.[45]

We calculate median fluxes for landfills with data from the GHGRP and the Landfill Methane Outreach Program grouped by waste type (municipal or industrial), whether there is gas collection infrastructure present, and whether the landfill is fully capped. We determine the presence of gas collection infrastructure and caps for the Facility Registry

Service landfills by visual inspection of Google Maps satellite images[50] and apply the relevant flux estimate to these landfills.

S1.4 Wastewater

S1.4.1 Treatment Plants

We use the following equation from the IPCC's inventory guidelines[51] to estimate methane emissions from population:

$$E_{CH_4} = P \times BOD \times B_o \times MCF \tag{2}$$

where P is population, BOD is biochemical oxygen demand, estimated at 8.5 g BOD person^{-1} day^{-1} (IPCC guidelines Table 6.4), B_o is maximum methane producing capacity, estimated at 0.6 kg CH_4 kg^{-1} BOD (IPCC guidelines Table 6.2), and MCF is the fraction of wastewater organic matter converted anaerobically, estimated at 0.05 (NYSDEC Sectoral Report 4: Waste[46]).

Since the NYSDEC Statewide GHG Report does not estimate separate weekday and weekend emission totals, we combine these to determine a scaling factor to match the emission totals in the NYSDEC Statewide GHG Report for centralized wastewater treatment and septic systems. For each subcategory, we first sum together our weekday and weekend emission estimates, weighting them by 5/7 and 2/7, respectively, to maintain spatial variations and estimated weekday-to-weekend emission ratios. We then calculate a scaling factor such that the total of this annual mean emission map matches the total emissions in the NYSDEC Statewide GHG Report. We then apply the scaling factor separately to the weekday and weekend gridded emission maps. For wastewater treatment plants, we then reduce emissions from treatment plants serving more than 50,000 people by 10 %, providing us with the emission totals to be distributed to the associated sewer systems.

S1.4.2 Sewers

We made Freedom of Information Law requests for "shapefiles showing either the locations of sewers or the areas served by wastewater treatment plants ("sewersheds")". Only Schenectady County provided the locations of sewer lines; all other counties that responded with data provided sewersheds. Suffolk County failed to provide us with a more complete dataset than the one publicly available in response to our Freedom of Information Law request. As a result, we do not distribute any emissions to sewers for 10 of the 30 treatment plants in Suffolk County. Rensselaer County does not host public sewershed data and did not respond to our Freedom of Information Law request; we therefore distribute 100 % of treatment plant emissions in the city of Troy as described in Section 2.4.1 of the main text.

We filter the TIGER roads dataset as described in Section S1.2.2 to exclude large highways or unpaved roads when distributing sewer emissions to sewersheds.

S1.4.3 Septic Systems

We disaggregate septic systems emissions first to the city, town, or village with which the population is associated in the Clean Watersheds Needs Survey where this information is available. For populations with no smaller municipality listed than county, we distribute to county and watershed using 8-digit hydrologic unit codes listed in both the Clean Watersheds Needs Survey and the US Geological Survey National Hydrography Dataset.[52] Finally, we distribute emissions to less-developed areas using grid cells in the National Land Cover Database[5] labeled "Developed - Open Area" or "Developed - Low Intensity," as we assume these areas are most likely to contain septic systems. As mentioned in the main text, estimated emissions based on the Clean Watershed Needs Survey data accounts for 90 % of the septic system emissions reported in the NYSDEC Statewide GHG Report and includes municipalities covering the majority of the state. To distribute emissions not accounted for by the emissions estimate based on the Clean Watershed

Needs Survey data, we assume the areas not included in this dataset ("remaining areas") are less densely populated than those that are included in the Clean Watersheds Needs Survey, and distribute to these remaining areas the remaining 10 % of emissions from the NYSDEC Statewide GHG Report. We first estimate emissions by applying the mean value of county emissions per applicable National Land Cover Database cell to these areas to account for spatial variety in population density in the remaining areas, and then scale the emissions calculated in this way to match the total remaining emissions.

S1.5 Other

S1.5.1 Stationary Combustion

We calculate monthly scaling factors for all natural gas stationary combustion emissions using monthly natural gas delivery by consumer type (residential, commercial, industrial, and electric, in Tables 14 through 17 of EIA Natural Gas Monthly[43]) from the Energy Information Administration as described in Section S1.2.3 above.

For stationary combustion emissions from power plants, we use the EPA's Power Sector Data Crosswalk,[53] which merges the EPA's Clean Air Markets Program Data[54] with US Energy Information Administration reports[55,56] to form a single electric power sector database. We use the maximum hourly fuel input reported in the Clean Air Markets Program Data for each fuel category, emission factors from Annex 3A of the EPA GHGI,[45] and the GHGRP-reported emissions by fuel type to estimate maximum hourly methane emissions. When multiple fuel types are used by a single power plant, we assign the GHGRP emissions proportionally using the maximum hourly methane emission estimates calculated as described above for each fuel type. For methane emissions from the energy sector combustion of coal, distillate fuel, and residual fuel, the sum of the GHGRP estimated emissions in New York exceed the Energy Sector GHG Report reported state emission totals. We do not use the GHGRP-reported emissions for energy sector consump-

tion of those fuel types and instead replace them with emissions estimated as described. New York only has one electricity generation facility that uses wood fuel, and its emissions are reported to the GHGRP. We replace the GHGRP-reported emissions with the Energy Sector GHG Report total for this facility. For the remaining fuel type, natural gas, we assign the GHGRP emissions to reporting facilities and scale the remaining emissions to match the Energy Sector GHG Report totals.

To estimate emissions using reported maximum fuel input from the U.S. Environmental Protection Agency (EPA)'s Clean Air Markets Program Data and emission factors from the EPA, we start with the Power Sector Data Crosswalk database merged with the original Clean Air Markets Program database to regain some of the additional attributes of Clean Air Markets Program Data, including "Max Hourly HI Rate (mmBtu/hr)."[54] The Clean Air Markets Program Data Glossary describes this attribute as "The design heat input capacity (mmBtu/hr) for the unit or the highest hourly heat input rate observed in the past five years, whichever is greater."[54] We use these values and fuel type to estimate maximum hourly methane emissions from each fuel type for each power plant based on EPA GHGI Annex 3A emission factors. From these maximum hourly methane emission values and the emission estimates from the GHGRP,[57] we back-calculate an estimate of annual maximum-capacity-equivalent runtime for GHGRP-reporting power plants. As the distribution is highly skewed, we apply the median of these estimated annual runtimes to non-reporting power plants to estimate methane emissions for those plants.

For residential and nonresidential natural-gas combustion, we distribute emissions only to tax parcels with natural-gas services or unlisted utility services within natural-gas service areas. For methane emissions from fuels besides natural gas, we include properties within natural-gas service areas, but we assume that properties with natural-gas service listed in the Tax Parcels database will have negligible combustion emissions for other fuels and exclude these properties.

We distribute residential stationary combustion emissions within counties to loca-

tions in the Tax Parcels database[38] using housing unit count estimated as described in Section S1.2.3. Nonresidential combustion is separated into commercial/institutional combustion and industrial combustion in the NYSERDA Energy Sector GHG Report. We distribute these emissions separately using the same method. We separate properties into these groups using property class fields as described in Section S1.2.3, and distribute emissions for each fuel type within counties by assessed property value listed in the Tax Parcels database.

S1.5.2 Mobile Combustion

The Federal Highway Administration's Highway Statistics 2020[58] reports vehicle miles traveled by road type, separated into urban and rural. The TIGER Roads database[30] database MAF/TIGER Feature Class Codes are also indicators of road type but have fewer categories. We group the Federal Highway Administration road types into MAF/TIGER Feature Class Codes based on MAF/TIGER Feature Class Codes descriptions. Table S6 shows the grouping used.

Table S6: The MAF/TIGER Feature Class Codes (MTFCC) from the Topographically Integrated Geographic Encoding and Referencing system (TIGER) Roads database[30] we assign to Federal Highway Administration road types to allocate vehicle miles traveled to TIGER Roads features.

**MTFCC**	**FHA road types**
S1100	INTERSTATE<br>OTHER FREEWAYS AND EXPRESSWAYS
S1200	OTHER PRINCIPAL ARTERIAL<br>MINOR ARTERIAL
S1400	MAJOR COLLECTOR<br>MINOR COLLECTOR<br>LOCAL

Table S7 shows the land types from the National Land Cover Database (NLCD)[5] we use to distribute off-road mobile emission categories from the Energy Sector GHG Report. For mobile emissions from recreational vehicles and miscellaneous non-road sources, we

apply a weighted distribution by city and town populations using the New York State Civil Boundaries database.[34] We also distribute non-road recreational vehicle emissions to state-owned lands from the New York Protected Areas Database.[7]

Table S7: Land types from the National Land Cover Database (NLCD) used in our distribution of selected off-road mobile combustion emission categories. Off-road mobile combustion emission categories not shown in this table are distributed using other data (see main text Section 2.5.3).

**Energy Sector GHG Report category**	**NLCD index**
Agricultural	82[a] ("cropland")
Construction	21 ("developed-open")<br>22 ("developed-low intensity")<br>23 ("developed-medium intensity")<br>24 ("developed-high intensity")
Lawn and Garden	21 ("developed-open")<br>22 ("developed-low intensity")<br>23 ("developed-medium intensity")
Marine / Boating	11 ("open water")
Nonroad - Miscellaneous/Unclassified	21[b] ("developed-open")
Nonroad - Other	21[b] ("developed-open")
Nonroad - Public Nonhighway	21[b] ("developed-open")
Recreational Vehicles	21[b,c] ("developed-open")

[a] Park areas excluded using the New York Public Areas Database.[7]
[b] Population-weighted distribution.
[c] Also distributed to state-owned lands using the New York Public Areas Database.[7]

S2 Data Limitations

The activity data we use has some limitations in its ability to represent emission sources. Here, we discuss these limitations and any impacts they may have on our results.

S2.1 Agriculture

Our distribution of agricultural methane emissions using "grassland / pasture" area from the satellite-based National Land Cover Database,[5] in addition to taking on any errors in the algorithm used to generate the dataset, also overlooks the significant contribution of

animal feeding operations (AFOs) to livestock populations and therefore emissions. This means that our distributed agriculture emissions are biased low in areas that have large livestock populations in animal feeding operations, and biased high elsewhere. The use of estimated animal population as spatial activity data for livestock methane emissions, even when spatially accurate, also means that our distribution of emissions from manure management are less accurate than those from enteric fermentation because manure is typically collected and stored separately from livestock in animal feeding operations.[59]

S2.2 Fossil Fuel Systems

Although we distribute natural gas distribution pipeline fugitive emissions to roads within natural gas service areas, we note that the total length of these roads is an order of magnitude smaller than the estimated total length of distribution pipelines in New York, as determined by dividing distribution pipeline emissions from the NYSERDA Oil and Gas Methane Inventory[26] by the emission factor per unit length published in the same report. This introduces error in distribution patterns within each gas service territory, as it is unclear whether the ratio of total road length to total pipeline length in a service area can be assumed constant, particularly between urban and rural areas.

S2.3 Solid waste

To estimate emissions for landfills not included in the GHGRP[57] or the Landfill Methane Outreach Program,[49] we use median fluxes calculated from those that are included in one or both of those datasets. The GHGRP only requires reporting from very large GHG sources, and the landfills included in the Landfill Methane Outreach Program but not the GHGRP have been involved in landfill gas collection projects. Inclusion in these datasets may therefore be biased towards larger landfills, meaning the application of their median emission flux to landfills excluded from the GHGRP and the Landfill Methane Outreach

Program may lead to an overestimation of the contribution of excluded landfills to the state total.

Several recent studies indicate that the GHGRP's bottom-up methodology produces values that are poorly correlated with observations and retrievals.[60–65] Our use of emission totals reported in the GHGRP and our use of the same equation as the GHGRP[45,57] to estimate emissions from waste-in-place may, therefore, to lead to discrepancies in the proportion of total New York landfill emissions allocated to each landfill.

S2.4 Wastewater

The use of only population reported by the EPA's 2012 Clean Watersheds Needs Survey to estimate wastewater treatment emissions ignores the potentially significant emissions impacts of the technology in use at each wastewater treatment facility and changes in population over time. Furthermore, the COVID-19 pandemic and the associated statewide stay-at-home order, mandated in New York by executive order effective March 22, 2020 and lifted to varying degrees by county based on reported infection statistics,[66] likely also impacted the spatial distribution and magnitude of wastewater emissions. For the duration of the order, wastewater emissions would likely have been closer to the resident ("weekend", as described in the main text) distribution. Furthermore, even after official reopening, spatially variable changes in population and in relative proportions of residents and non-residents may have persisted, adding to any such changes that have occurred since the 2012 release of the Clean Watersheds Needs Survey dataset.[67] These changes are not captured by the spatial distribution of wastewater emissions.

S2.5 Other

Our distribution of stationary combustion methane emissions in the energy sector fails to account for the use of multiple fuel types in a single boiler, as we lack records of

consumption of every fuel type at each energy facility or each boiler within facilities. This means that some power plants may not have their emissions from all fuel types represented in the gridded inventory.

We distribute residential, commercial, and industrial stationary combustion methane emissions, from nearly all fuel types equally to all buildings of the respective type statewide. Only natural gas combustion emissions are distributed to a subset of buildings, namely those within gas service areas that do not indicate a lack of gas utility service. The same subset is used for fugitive emissions from gas meters and end use. This method errs on the side of broader distribution of these emissions in the interest of facilitating comparison with observed emissions using inverse methods.

S3 Uncertainty

Table S8 shows relative uncertainties for each emission category. To apply the Maasakkers *et al.*[4] uncertainty equation (Equation 1 in the main text), we approximate 100 m resolution as $\tau = 0.001^\circ$, as a 100 m$\times$100 m grid cell is approximately $0.0012^\circ \times 0.0009^\circ$ at the latitude of New York. Although methane emissions from gas end-use fugitives and waste combustion are estimated in the 2022 EPA GHGI, individual uncertainty estimates for these source categories are not provided in Annex 7: Uncertainty. The 2018 GEPA2 inventory data also does not include natural gas end-use fugitives and waste combustion. Since the 2018 data is the one used by Maasakkers *et al.*[4] to generate resolution-dependent relative uncertainties and error decay coefficients (σ_R, k_τ), these are not explicitly included in the GEPA2 inventory. We apply the national relative uncertainties (σ_N), resolution-dependent relative uncertainties (σ_R) and error decay coefficients (k_τ) of stationary combustion and landfill emissions, respectively, to these emission categories with the assumption that they have the most similar uncertainties in both aggregate totals and spatial distribution.

Table S8: Total relative uncertainties (σ) for each emission category at τ =0.1° and τ =100 m, broken down into statewide total (σ_N) and resolution-dependent (σ_R, σ_τ) components, and error decay coefficients (k_τ). Note that at $\tau = 0.1$, $\sigma_\tau = \sigma_R$.

				$\tau = 0.1°$		$\tau = 100$ m	
Category	IPCC	σ_N	k_τ	σ_R	σ	σ_τ	σ
Agriculture	4						
Enteric fermentation	4A	14.5%	3.12	88.0%	**102.5%**	119.8%	**134.3%**
Manure management	4B	19.0%	3.12	88.0%	**107.0%**	119.8%	**138.8%**
Fossil fuel systems	1B						
Oil systems	1B2a	30.0%	0.71	38.0%	**68.0%**	40.8%	**70.8%**
Gas systems	1B2b1,2	18.0%	0.13	44.0%	**62.0%**	44.6%	**62.6%**
Gas end-use fugitives	1B2b3	79.5%	10.86	32.0%	**111.5%**	93.8%	**173.3%**
Abandoned wells	1B2d	140.0%	0.71	38.0%	**178.0%**	40.8%	**180.8%**
Solid waste	6A,C	22.5%	4.02	19.0%	**41.5%**	28.3%	**50.8%**
Wastewater	6B	29.0%	10.86	32.0%	**61.0%**	93.8%	**122.8%**
Other	1A, 2C						
Iron & steel industry	2C	22.0%	4.02	19.0%	**41.0%**	28.3%	**50.3%**
Stationary combustion	1A1,2,4	79.5%	10.86	32.0%	**111.5%**	93.8%	**173.3%**
Mobile combustion	1A3	16.0%	10.86	32.0%	**48.0%**	93.8%	**109.8%**

S4 Detailed Comparisons

Here we provide additional details on the comparisons described in Section 3.1 of the main text.

S4.1 The Gridded EPA Inventory (GEPA2) and the Emissions Database for Global Atmospheric Research version 8.0 (EDGARv8)

Figure S1 shows NY methane emission fluxes in the GEPA2 and EDGARv8 inventories and their differences with this work. Additional figures comparing emissions for each category in this work with those of the GEPA2 inventory and the EDGARv8 inventory are available in Section S6.2 and S6.3, respectively.

The much larger total estimated agricultural methane emissions in this work relative to the EDGARv8 emissions, shown in Table 1 of the main text, appear in Figure S17 as widespread regions of positive bias in our work relative to the EDGARv8 emissions, while

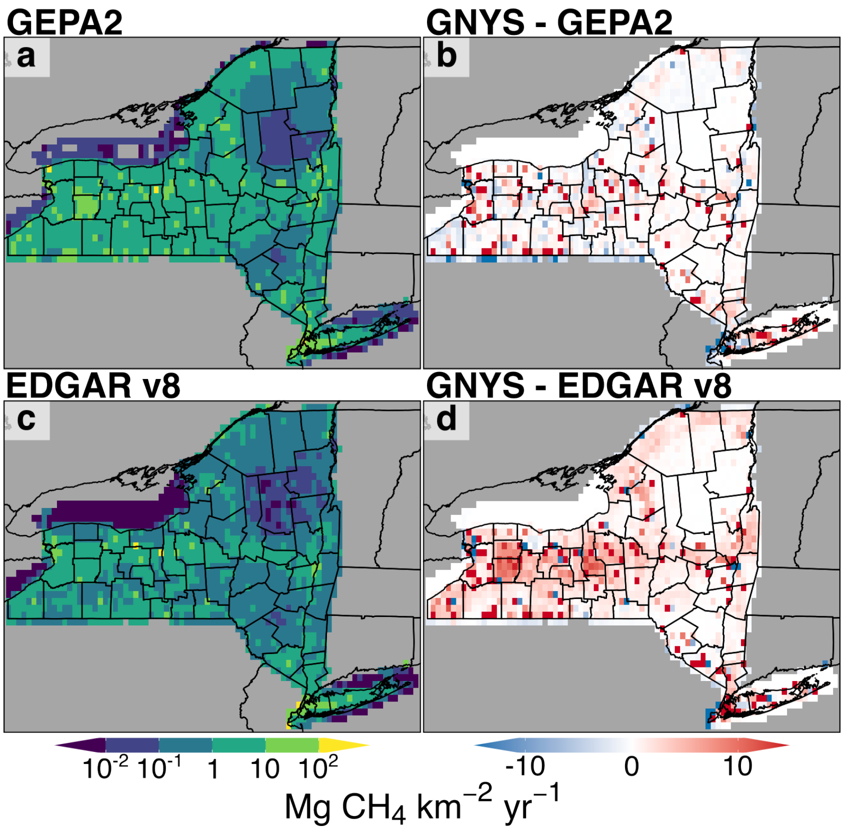

Figure S1: Comparison between gridded total anthropogenic methane emission fluxes. (a, c) Fluxes in NY only in the GEPA2[4] and the EDGARv8[68] inventories, respectively. (b, d) the difference between the NY emission fluxes from the gridded NY inventories (GNYS), remapped to 0.1° horizontal resolution, and those from the GEPA2 and the EDGARv8 inventories, respectively. We use tools from the Geospatial Data Abstraction Library (GDAL)[18] and Climate Data Operators (CDO)[69] to perform remapping. Black lines show state/provincial and NY county borders. Note that the color scale for subplots (a, c) is logarithmic while the color scale for subplots (b, d) is linear.

agricultural emission totals are nearly identical in the NY and the GEPA2 inventories. Agricultural emissions are distributed with very similar patterns statewide in all three inventories (Figures S12, S17).

The distribution of fossil fuel production emissions across a greater area in the GEPA2 inventory relative to this work is visible in Figure S1 as an underestimation of gridded emissions in our work relative to the GEPA2 emissions in western NY. This is due to differences in emission factors and disaggregation methods for active gas wells which make up the majority of emissions from fossil fuel systems in this area.

In this work, we allocate greater wastewater emission flux to NYC and lesser to Long Island and southern Westchester County relative to both the GEPA2 and the EDGARv8 inventories (Figures S15, S20). The GEPA2 inventory distributes wastewater emissions using EPA-reported flow rates and capacities for treatment plants and using population for septic systems,[4] while the EDGARv8 inventory distributes using population for all wastewater emissions using the approach of Bartram *et al.*[51,70] This work, however, differentiates between resident and non-resident populations as described in Section 2.4 of the main text, leading to greater emission flux within NYC and lesser in the surrounding area. This effect is more pronounced when comparing to the EDGARv8 emissions than to the GEPA2 emissions as the EDGARv8 inventory distributes all wastewater emissions by population and allocates greater emissions to wastewater.[71]

The spatial allocation of gas distribution emissions in NY by population in the GEPA2 inventory leads to greater emissions in NYC relative to this work, visible in Figure S1. These emissions are also distributed by population in the EDGARv8 inventory, but the differences are smaller due to lower total emissions in this category.

Although the absolute emission totals are too small for the difference to be visible in Figure S1, the GEPA2 marine and boating emissions are distributed to geographic data showing navigable waterways from the US Department of Transportation,[72] leading to the different patterns of emissions in Lake Ontario in Figure S16A and B.

Table S9 shows statistical comparisons between emissions in New York in 2020 in this work (remapped to 0.1° resolution), the updated gridded EPA inventory Express Extension (GEPA2), and the Emissions Database for Global Atmospheric Research version 8.0 (EDGARv8). Agriculture and "other" emissions compare favorably with both the GEPA2 and EDGARv8 inventories by all metrics. Correlation between this work and the GEPA2 and EDGARv8 inventories are substantially different in the solid waste category due to the same spatial error in the EDGARv8 solid waste inventory mentioned in Section 3.1.1 of the main text. The solid waste and fossil fuel systems categories have relatively higher mean

biases and RMSE primarily due to these two categories dominating the large differences in total emissions between the gridded inventories as shown in Table 1 of the main text. Poor correlation in the wastewater category is a result of the differences in spatial disaggregation techniques mentioned in Section 3.1.1 of the main text. Our work also shows poor correlation with the EDGARv8 inventory in the fossil fuel systems category. The EDGARv8 inventory uses population data to distribute methane emissions from compressor stations, one of the largest point sources of methane in this work.[26,68,70] Furthermore, in western New York, where active oil and gas wells dominate methane emissions, the EDGARv8 inventory shows more evenly-distributed emissions from fossil fuel systems relative to this work and the GEPA2 inventory (differences shown in Figures S18 and S13). The EDGARv8 inventory uses a per-well emission factor for active wells,[37,68,70] while we reproduce the methods of the Oil and Gas Methane Inventory in our use of production-dependent emission factors.[26] The GEPA2 inventory, like this work, distributes well-pad emissions in New York based on well-level production data and uses proprietary data for compressor station locations.[4] As a result, spatial patterns of methane emissions from fossil fuel production in the GEPA2 inventory and this work are very similar despite the large differences in total emissions.

We compare fossil fuel systems emissions in this work with those of the GEPA2 inventory due to its greater transparency in methods and more numerous subcategories than the EDGARv8 inventory. Compressor stations are some of the largest sources of fossil methane in the NYSERDA Oil and Gas Methane Inventory. The NYSERDA inventory and the EPA GHGI, which underlies the GEPA2 inventory, both use per-compressor-station emission factors from Zimmerle *et al.*,[16,26,73] while the Emissions Database for Global Atmospheric Research inventory version 8.0 (EDGARv8) inventory uses production-based emission factors for transmission and storage from the IPCC.[37,70] However, the Oil and Gas Methane Inventory estimates the number of transmission compressor stations in New York at one every 70 miles of transmission pipeline, calculating a total of 64 transmission compressor

Table S9: Spatial correlation coefficients, mean biases, and root mean square error (RMSE) between this work, conservatively remapped to 0.1° horizontal resolution, and two existing gridded methane inventories at 0.1° horizontal resolution: the GEPA2 Express Extension for 2020[4] and the EDGARv8 inventory.[68] Values are calculated assuming proportional distribution by area for grid cells in the GEPA2 and the EDGARv8 inventories that cross the New York border.

			$Mg\ CH_4\ km^{-2}\ yr^{-1}$	
	**Category**	**Correlation coefficient**	**Mean bias**	**RMSE**
GEPA2	Agriculture	0.91	0.20	1.2
	Fossil fuel systems	0.50	0.41	5.9
	Solid waste	0.83	0.95	6.0
	Wastewater	0.28	0.083	0.55
	Other	0.81	0.014	0.29
	Total	0.73	1.7	9.0
EDGARv8	Agriculture	0.88	1.4	1.5
	Fossil fuel systems	0.18	0.84	3.2
	Solid waste	-0.0017	0.97	13
	Wastewater	0.20	0.011	1.6
	Other	0.90	-0.014	0.54
	Total	0.076	3.2	15

stations in New York. The EPA GHGI estimates national compressor station counts by starting with the number of GHGRP-reporting stations and applying a scaling factor to account for non-reporting stations.[16] The Rextag data product indicates only 24 transmission compressor stations statewide;[74] as a result, this work assigns more than double the emissions to each compressor station. Submitted work by Ravikumar *et al.*[75] contains not only a more reliable count of compressor stations in New York (41 transmission, 21 storage, 17 gathering, and 1 distribution compressor stations) than Rextag or the estimate in the Oil and Gas Methane Inventory, but also measurement-based emission factor estimates and corresponding statewide emissions that are much lower than those reported by the Oil and Gas Methane Inventory. Updating the station counts and emission factors for compressor stations with the values from Ravikumar *et al.*[75] would account for almost the entire difference in fossil fuel systems totals between the NYSERDA inventory and the GEPA2 New York total. Although the NYSERDA inventory uses as a baseline the same activity

data and emission factors as the GEPA2 inventory for fugitive methane emissions from distribution pipelines, it estimates a total of 44 Gg yr^{-1} from this source in 2020 in New York, 9 Gg yr^{-1} higher than the 2020 estimate from the GEPA2 inventory, most likely due to the NYSERDA inventory's update to the emission factors based on GHGRP-reported emissions from utility companies in New York. Although upstream emissions from oil in New York are much smaller than those from gas, the GEPA2 inventory notably allocates more than four times as much methane to this category (7.9 Gg CH_4 yr^{-1} in 2020) as the NYSERDA Oil and Gas Methane Inventory (1.7 Gg CH_4 yr^{-1} in 2020). We are unable to compare the underlying spatial data used for distribution of these emissions due to the use of a proprietary dataset for well locations and production volumes in the GEPA2 inventory. However, the majority of upstream oil emissions in the EPA GHGI come from sources that use per-well emission factors that may not be representative of New York, while the NYSERDA inventory uses production-based emission factors calculated for the Appalachian region.[16,26]

Figure S2 compares monthly NY methane emissions in this work (GNYS) with those of the EDGARv8[68] and GEPA2[4] inventories for categories with monthly variability. We plot data from 2018 for the GEPA2 inventory as this is the latest year for which GEPA2 has full seasonality.

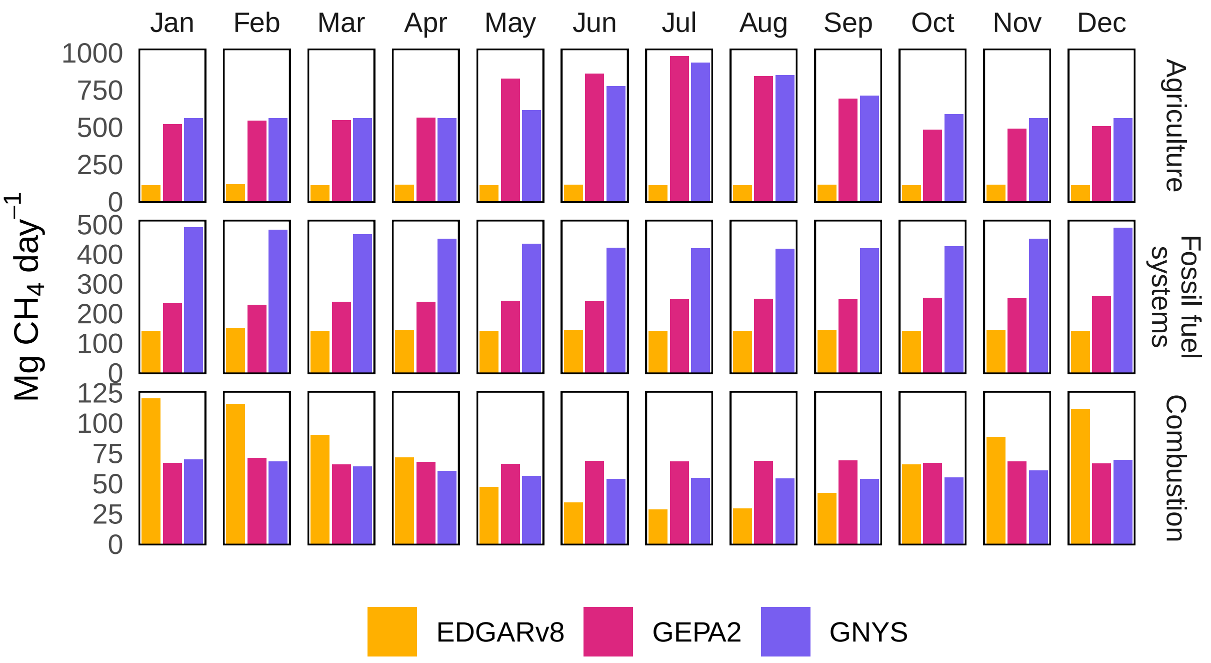

Figure S2: Bar plots of monthly methane emissions in NY in the GEPA2 inventory (2018 data), the EDGARv8 inventory (2020 data), and this work (GNYS; 2020 data) for the three emission categories with monthly variability.

S4.2 The New York City Urban Area Inventory (NY-UA)

Figure S3 shows emissions in this work and the NY-UA inventory in the overlap of their domains, as well as their difference. Figures matching Figure S3 comparing the different categories shown in Table 2 of the main text can be found in Section S6.4.

The NY-UA inventory versions HRA, HRB, HRC, and HRD differ in the spatial extents used to calculate some emission totals as well as in some of the data sources used to distribute emissions.[76] These differences are summarized in Table S10 (adapted from *et al.*[76]).

This work includes more gas transmission pipelines than the NY-UA inventory, as we use a more complete private dataset[74] for pipeline locations, although these contribute little to total transmission emissions. We exclude emissions from natural-gas distribution and post-meter fugitives from areas listed by the New York State Department of Public Service[31] to be without gas service, while the NY-UA inventory does not. However,

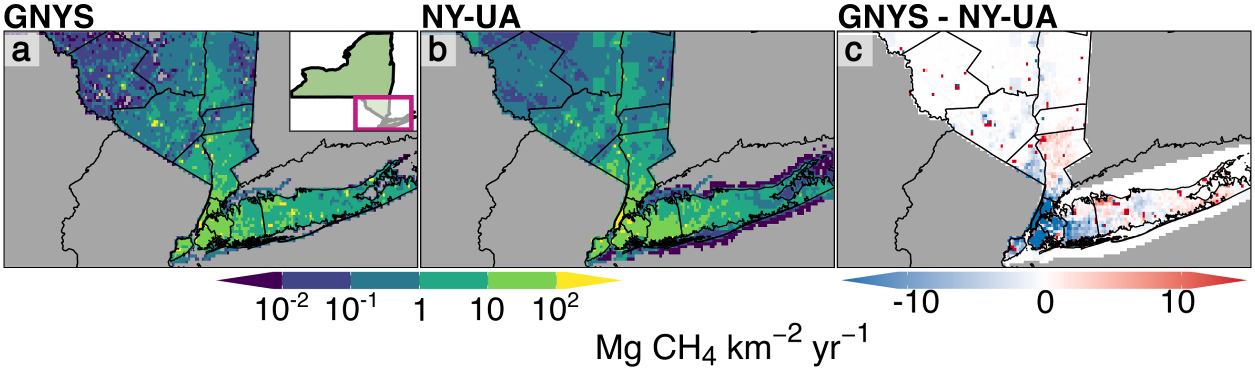

Figure S3: Comparison between anthropogenic methane emission fluxes from landfills, natural-gas midstream and downstream infrastructure, stationary fuel combustion, and wastewater in two gridded inventories. (a, b) Fluxes in this work (GNYS) and gridded inventory version HRB from Pitt *et al.*[76] (NY-UA), respectively, in the overlap in their domains. (a) Emissions are remapped to 0.02° horizontal resolution using command-line tools from the GDAL[18] and CDO[69] libraries. (c) the difference between (a) and (b). Inset map of NY in (a) indicates the area plotted, the overlap in the domains of the NY-UA inventory and this work. Black lines show state and NY county borders. Note that the color scale for subplots (a, b) is logarithmic while the color scale for subplot (c) is linear.

the NY-UA inventory assigns relatively low emission fluxes to these areas, even in the versions of their gridded inventory that do not distribute emission totals within local gas distribution company territories. As a result, the spatial pattern of gas distribution and post-meter fugitive emissions in this work is still very similar to that of the NY-UA inventory (Figures S23, S24).

Emissions of methane from natural-gas distribution systems in our work and the New York City Urban Area (NY-UA) inventory compare well as a result of the similarities in spatial disaggregation method. The NY-UA inventory uses emission factors from Weller *et al.*,[82] while we use emission factors from the Oil and Gas Methane Inventory based on the work of Lamb *et al.*[83] and scaled to approximately match data reported by utilities.[26] Both the NY-UA inventory and this work then use data from the Pipelines and Hazardous Materials Safety Administration[32] to calculate emissions by pipeline type and operator. Version HRB of the NY-UA inventory distributes emissions to operator areas using residential and commercial fossil fuel combustion CO_2 emission maps from the Anthropogenic Carbon Emission System v2[77] (ACES v2). In this work, we distribute

Table S10: Different data sources, spatial proxies, and emission calculation extents used in the different versions of the New York City Urban Area (NY-UA) inventory.[76] For gas distribution & end use, stationary combustion, and septic systems, the "Emissions" row indicates the level at which emissions were aggregated prior to distribution. For wetlands, the "Emissions" row indicates the source used for wetland emission fluxes. The "Distribution" row indicates the dataset used to distribute emissions. Further details are available in *et al.*[76]

		NY-UA version			
**Emission category**		**HRA**	**HRB**	**HRC**	**HRD**
**Gas distribution**	Emissions	State	LDC[a] [a]	5-state	LDC
**& end-use**	Distribution	ACES[b] v2[77]	ACES v2	Vulcan[78]	Vulcan
**Stationary**	Emissions	State	State	State	State
**combustion**	Distribution	Vulcan	ACES v2	Vulcan	Vulcan
**Septic systems**	Emissions	National	State	State	State
**Wetlands**	Emissions	WetCHARTs[79]	WetCHARTs	SOCCR1[c] [80]	SOCCR1
	Distribution	NLCD[5]	NLCD	NWI[d] [81]	NWI

[a] Local Distribution Company [b] Anthropogenic Carbon Emission System [c] State of the Carbon Cycle Report [d] National Wetlands Inventory
[a] Local distribution company.

emissions in each operator area by road length as a proxy for pipeline length as described in Section 2.2.2 of the main text.

This work and the NY-UA inventory both distribute stationary combustion emissions using the EPA National Emissions Inventory[84] data on CO emissions by county, fuel type, and consumer type. The use of identical county-level activity data in the two gridded inventories indicates that the differences in stationary combustion emission totals for this area are a result of emission factors. The NYSERDA Energy Sector GHG Report uses US-specific emission factors from the EPA,[36] while the NY-UA inventory uses emission factors from the IPCC[37] and Hajny *et al.*[85]

The use of wastewater flow rates from the Clean Watersheds Needs Survey[67] in the NY-UA inventory, unlike the use of the EPA flow rates and capacities in the GEPA2 inventory,[4] does not produce a large difference in total emissions compared to this work, and the NY-UA inventory distributes greater wastewater emissions to the area considered

Table S11: Grouping of source types from this work (GNYS) into the categories of the NY-UA inventory

NY-UA category	Source type(s)
Landfills	Landfills
Natural-gas distribution	Distribution pipelines<br>Gas meters (all sectors)
Natural-gas post-meter	Residential appliances<br>Residential buildings<br>Residential gas combustion
Natural-gas transmission	Transmission pipelines<br>Transmission compressor stations
Stationary combustion - fossil fuels	All stationary combustion except<br>wood (all sectors) & residential natural gas
Stationary combustion - wood	Wood combustion (all sectors)
Wastewater	Treatment plants<br>Sewers<br>Septic systems

here than the GEPA2 inventory does to the entire state. Since our work also uses data from the Clean Watersheds Needs Survey, this highlights differences between the Clean Watersheds Needs Survey and the EPA wastewater flow datasets as well as uncertainty in the calculation of wastewater emissions.

Table S12 shows spatial correlation coefficients, mean biases, and root mean square error (RMSE) between emission fluxes from this work (GNYS; conservatively remapped to 0.02° resolution as above) and the NY-UA inventory version HRB for each emission category considered in Table 2 of the main text within the overlap of their domains as well as for total emission fluxes in that area. Comparison statistics for the total inventory are quite good, and statistics for individual categories show that contrasts are concentrated primarily in categories with large differences in total emissions. Landfill emissions are reasonably well correlated due to the use of locations and emissions from the GHGRP in both inventories, but this similarity is limited by the differences in spatial distribution method and non-GHGRP emission estimates in the two inventories as mentioned above. Higher correlations in fluxes from gas infrastructure reflect the robustness of the different

techniques used to distribute these emissions within the domain considered here. However, as mentioned above, observations from several studies suggest that these emissions are too low in both inventories.[63,76,82,86–88] Our distribution of wastewater treatment emissions to sewers in urban areas leads to higher absolute mean bias and RMSE relative to the NY-UA inventory, which distributes all centralized treatment emissions to treatment plants using municipal flow rates reported in the Clean Watersheds Needs Survey. However, because both inventories distribute emissions from septic systems to less-developed areas from the National Land Cover Database,[5] correlation is still very high.

To calculate the values shown in Table 2 of the main text and Tables S12 through S18 here, we grouped individual source types from this work into the categories used in the NY-UA inventory, then conservatively remapped them to 0.02° horizontal resolution to match the native resolution of the NY-UA inventory. Finally, we applied a New York mask to the NY-UA gridded emission maps and calculated the totals of each gridded inventory map.

Table S12: Spatial correlation coefficients, mean biases, and root mean square error (RMSE) between this work, conservatively remapped to 0.02° horizontal resolution, and inventory version HRB of the New York City Urban Area inventory (NY-UA) from Pitt *et al.*[76] "Total" includes only the source categories and categories listed here; NY-UA emission categories for which gridded emissions are taken directly from the GEPA2 inventory are excluded. Values are calculated assuming proportional distribution by area for grid cells in the NY-UA inventory that cross the New York border.

**Category**	**Correlation coefficient**	Mg CH_4 km^{-2} yr^{-1} **Mean bias**	**RMSE**
Landfills	0.42	0.01	29
Natural-gas distribution	0.73	-0.79	3.6
Natural-gas post-meter [a]	0.92	-1.4	4.7
Natural-gas transmission	0.62	0.05	13
Stationary combustion - fossil fuels	0.78	-0.16	1.0
Stationary combustion - wood	0.88	-0.14	0.36
Wastewater	0.94	-0.69	9.0
Total	0.49	-3.1	34

[a] Residential only.

Tables S13 through S18 compare methane emissions by category from versions HRA, HRC, and HRD of the NY-UA inventory and this work (GNYS) in the overlap of their domains, matching Table 2 (main text) and Table S12 which compare with NY-UA inventory version HRB. The NY-UA inventory estimates emissions using 2019 activity data while the GNYS inventory uses New York inventories developed for 2020.[26,89,90]

Table S13: Anthropogenic methane emissions by category from version HRA of the New York City Urban Area Inventory[76] (NY-UA) and this work (GNYS) in the overlap of their domains. "Total" includes only the source categories listed here. For the NY-UA inventory, values outside parentheses calculated assuming constant flux within grid cells that cross the New York border and are therefore proportional to cell area within New York borders. Inside parentheses, lower and upper bounds assume none or all of the emissions in those cells occur in New York, respectively.

	Methane emissions in Gg yr^{-1}	
**Category**	**NY-UA - HRA**	**GNYS**
Landfills	$17^{+0.0054}_{-.21}$	46
Natural gas distribution	$32^{+1.0}_{-1.2}$	32
Natural gas post-meter[a]	$26^{+0.97}_{-0.54}$	6.8
Natural gas transmission	$3.0^{+0.17}_{-0.67}$	11
Stationary combustion - fossil fuels	$4.3^{+0.49}_{-0.37}$	2.7
Stationary combustion - wood	$4.3^{+0.081}_{-0.17}$	3.9
Wastewater	$20.^{+0.98}_{-2.7}$	20.
**Total**[b]	$\mathbf{110}^{+6.9}_{-3.3}$	**130**

[a] Residential only. [b] Category subtotals do not sum to total due to rounding errors.

Table S14: Spatial correlation coefficients, mean biases, and root mean square error (RMSE) between this work, conservatively remapped to 0.02° horizontal resolution, and NY-UA inventory version HRA from Pitt *et al.*[76] "Total" includes only the source categories listed here. Values calculated assuming proportional distribution by area for NY-UA grid cells that cross the New York border.

		$Mg\ CH_4\ km^{-2}\ yr^{-1}$	
**Category**	**Correlation coefficient**	**Mean bias**	**RMSE**
Landfills	0.42	0.01	29
Natural gas distribution	0.79	-0.99	3.7
Natural gas post-meter	0.92	-1.2	3.1
Natural gas transmission	0.62	0.05	13
Stationary combustion - fossil fuels	0.76	-0.16	1.0
Stationary combustion - wood	0.86	-0.14	0.37
Wastewater	0.94	-0.60	9.0
Total	0.49	-3.0	34

Table S15: Anthropogenic methane emissions by category from version HRC of the New York City Urban Area Inventory[76] (NY-UA) and this work (GNYS) in the overlap of their domains. "Total" includes only the source categories listed here. For the NY-UA inventory, values outside parentheses calculated assuming constant flux within grid cells that cross the New York border and are therefore proportional to cell area within New York borders. Inside parentheses, lower and upper bounds assume none or all of the emissions in those cells occur in New York, respectively.

	Methane emissions in $Gg\ yr^{-1}$	
**Category**	**NY-UA - HRC**	**GNYS**
Landfills	$17^{+0.0054}_{-.21}$	46
Natural gas distribution	$43^{+1.1}_{-1.9}$	32
Natural gas post-meter[a]	$28^{+0.81}_{-0.86}$	6.8
Natural gas transmission	$3.0^{+0.17}_{-0.67}$	11
Stationary combustion - fossil fuels	$4.3^{+0.49}_{-0.37}$	2.7
Stationary combustion - wood	$4.3^{+0.081}_{-0.17}$	3.9
Wastewater	$22^{+0.70}_{-3.0}$	20.
**Total**[b]	$\mathbf{130^{+1.5}_{-9.6}}$	**130**

[a] Residential only. [b] Category subtotals do not sum to total due to rounding errors.

Table S16: Spatial correlation coefficients, mean biases, and root mean square error (RMSE) between this work, conservatively remapped to 0.02° horizontal resolution, and NY-UA inventory version HRC from Pitt *et al.*[76] "Total" includes only the source categories listed here. Values calculated assuming proportional distribution by area for NY-UA grid cells that cross the New York border.

Category	Correlation coefficient	Mg CH_4 km^{-2} yr^{-1} Mean bias	RMSE
Landfills	0.42	0.01	29
Natural gas distribution	0.80	-1.5	5.9
Natural gas post-meter[a]	0.94	-1.3	4.1
Natural gas transmission	0.62	0.05	13
Stationary combustion - fossil fuels	0.76	-0.16	1.0
Stationary combustion - wood	0.86	-0.14	0.37
Wastewater	0.94	-0.69	9.0
Total	0.48	-3.7	35

[a] Residential only.

Table S17: Anthropogenic methane emissions by category from version HRD of the New York City Urban Area Inventory[76] (NY-UA) and this work (GNYS) in the overlap of their domains. "Total" includes only the source categories listed here. For the NY-UA inventory, values outside parentheses calculated assuming constant flux within grid cells that cross the New York border and are therefore proportional to cell area within New York borders. Inside parentheses, lower and upper bounds assume none or all of the emissions in those cells occur in New York, respectively.

Category	Methane emissions in Gg yr^{-1} NY-UA - HRD	GNYS
Landfills	$17^{+0.0054}_{-.21}$	46
Natural gas distribution	$28^{+0.73}_{-1.4}$	32
Natural gas post-meter[a]	$31^{+0.39}_{-1.4}$	6.8
Natural gas transmission	$3.0^{+0.17}_{-0.67}$	11
Stationary combustion - fossil fuels	$4.3^{+0.49}_{-0.37}$	2.7
Stationary combustion - wood	$4.3^{+0.081}_{-0.17}$	3.9
Wastewater	$22^{+0.70}_{-3.0}$	20.
**Total[b]**	$\mathbf{110}^{+8.7}_{-1.7}$	**130**

[a] Residential only. [b] Category subtotals do not sum to total due to rounding errors.

Table S18: Spatial correlation coefficients, mean biases, and root mean square error (RMSE) between this work, conservatively remapped to 0.02° horizontal resolution, and NY-UA inventory version HRD from Pitt *et al.*[76] "Total" includes only the source categories listed here. Values calculated assuming proportional distribution by area for NY-UA grid cells that cross the New York border.

		Mg CH_4 km^{-2} yr^{-1}	
**Category**	**Correlation coefficient**	**Mean bias**	**RMSE**
Landfills	0.42	0.01	29
Natural gas distribution	0.77	-0.79	3.3
Natural gas post-meter[a]	0.93	-1.39	5.0
Natural gas transmission	0.62	0.05	13
Stationary combustion - fossil fuels	0.76	-0.16	1.0
Stationary combustion - wood	0.86	-0.14	0.37
Wastewater	0.94	-0.69	9.0
Total	0.48	-3.1	34

[a] Residential only.

S5 Source category tables

Tables S19 through S25 show emission totals from the inventories prepared by the New York State Department of Environmental Conservation (NYSDEC) and the New York State Energy Research and Development Authority (NYSERDA) as reported at https://data.ny.gov.

Table S19: Agricultural methane emission totals as reported by the New York State Department of Environmental Conservation (NYSDEC) Statewide Greenhouse Gas Emissions Report *Sectoral Report 3: Agriculture, Forestry, and Land Use.*[91] Categories in boldface indicate subtotals of the lines indented below them. Emissions are reported in Gg methane year^{-1}.

Category	Methane emissions
**Agriculture**	**239**
**Enteric fermentation**	**163**
Dairy cattle	127
Beef cattle	32.0
Sheep	8.33×10^{-1}
Goats	3.57×10^{-1}
Horses	2.74
Deer/elk	1.19×10^{-1}
**Manure**	**76.2**
Dairy cattle	72.0
**Chickens***	2.86
Distributed by population	2.83
Applied as fertilizer	2.86×10^{-2}
Beef cattle	7.14×10^{-1}
Swine	3.57×10^{-1}
Horses	2.38×10^{-1}

* Reported as a single emission category in the NYSDEC report but distributed separately in this work. See main text for details.

Table S20: Fossil fuel systems methane emission totals as reported by the New York State Energy Research and Development Authority (NYSERDA) *New York State Oil and Gas Methane Emissions Inventory*.[26] Categories in boldface indicate subtotals of the lines indented below them. Emissions are reported in Gg methane year^{-1}.

Category	Methane emissions
**Fossil fuel systems**	**168**
**Upstream**	**34.2**
**Gas well pads**	**32.3**
Low-producing	24.4
High-producing	7.88
**Oil well pads** [a]	**1.29**
Low-producing	1.28
High-producing	1.27×10^{-2}
Mud degassing	2.76×10^{-1}
Abandoned oil wells	1.92×10^{-1}
Well completions	7.99×10^{-2}
Abandoned gas wells	6.87×10^{-2}
Drilling fugitives	2.55×10^{-3}
Drill rigs	4.76×10^{-5}
**Midstream**	**117**
Distribution pipeline	44.5
Transmission compressor stations	42.9
Storage compressor stations	22.0
LNG [b] compressor stations	3.23
Transmission pipeline	2.81
**Gathering and processing**	**8.43**$\times10^{-1}$
Gas	8.22×10^{-1}
Oil	2.09×10^{-2}
Gathering pipeline	4.31×10^{-1}
**End-use fugitives**	**17**
Residential buildings	5.48
Residential meters	4.54
Commercial meters	2.65
Residential appliances	2.44
**Commercial buildings** [c]	**1.89**
Restaurants & hospitals	1.87
All others	1.89×10^{-2}

[a] Reported as separate emission categories in the NYSERDA report but distributed together in this work. See main text for details. [b] Liquefied natural gas. [c] Reported as a single emission category in the NYSERDA report but distributed separately in this work. See main text for details.

Table S21: Solid waste methane emission totals as reported by the NYSDEC Statewide Greenhouse Gas Emissions Report *Sectoral Report 4: Waste*.[46] Emissions from waste exported out of New York are not shown here. Categories in boldface indicate subtotals of the lines indented below them. Emissions are reported in Gg methane year^{-1}.

Category	Methane emissions
**Solid waste**	**233**
Landfills	232
Waste combustion	8.79×10^{-1}

Table S22: Wastewater methane emission totals as reported by the NYSDEC Statewide Greenhouse Gas Emissions Report *Sectoral Report 4: Waste*.[46] Categories in boldface indicate subtotals of the lines indented below them. Emissions are reported in Gg methane year^{-1}.

Category	Methane emissions
**Wastewater**	**30.8**
**Centralized treatment***	**16.2**
Treatment plants	15.0
Urban sewers	1.19
Septic systems	14.6

* Reported as a single emission category in the NYSDEC report but distributed separately in this work. See main text for details.

Table S23: Industrial methane emission totals as reported by the the NYSDEC Statewide Greenhouse Gas Emissions Report *Sectoral Report 2: Industrial Processes and Product Use*.[92] Categories in boldface indicate subtotals of the lines indented below them. Emissions are reported in Gg methane year^{-1}.

Category	Methane emissions
**Industry**	**1.31**$\times10^{-4}$
Iron and steel plants	1.31×10^{-4}

Table S24: Stationary combustion methane emission totals as reported by the NYSEREDA *Energy Sector Greenhouse Gas Emissions Report*.[90] Categories in boldface indicate subtotals of the lines indented below them. Emissions are reported in Gg methane year^{-1}.

**Category**	**Methane emissions**
**Stationary Combustion**	**17.2**
**Residential buildings**	**12.3**
Wood	8.91
Natural Gas	2.38
Distillate fuel	8.20×10^{-1}
Liquefied petroleum gas	1.35×10^{-1}
Kerosene	3.30×10^{-2}
**Commercial buildings**	**3.62**
Wood	1.59
Natural Gas	1.58
Distillate fuel	3.91×10^{-1}
Liquefied petroleum gas	5.01×10^{-2}
Residual Fuel	5.94×10^{-3}
Kerosene	3.20×10^{-3}
**Industrial buildings**	$\mathbf{7.53}\times10^{-1}$
Wood	5.69×10^{-1}
Natural Gas	8.81×10^{-2}
Coal	4.17×10^{-2}
Distillate fuel	2.79×10^{-2}
Petroleum Coke	1.52×10^{-2}
Kerosene	6.89×10^{-3}
Residual Fuel	3.87×10^{-3}
Special Naphthas	4.29×10^{-4}
Liquefied petroleum gas	2.62×10^{-4}
**Electricity generation**	$\mathbf{5.31}\times10^{-1}$
Natural Gas	4.61×10^{-1}
Wood	6.68×10^{-2}
Coal	1.21×10^{-3}
Residual Fuel	1.13×10^{-3}
Distillate fuel	9.76×10^{-4}
Fuel transportation via pipeline	2.92×10^{-2}

Table S25: Mobile combustion methane emission totals as reported by the NYSEREDA *Energy Sector Greenhouse Gas Emissions Report*.[90] Categories in boldface indicate subtotals of the lines indented below them. Emissions are reported in Gg methane year^{-1}.

**Category**	**Methane emissions**
**Mobile Combustion**	**4.66**
**On-road**	**3.39**
Compressed natural gas	1.63
Gasoline	1.51
Diesel	2.56×10^{-1}
Lawn and garden [a]	4.01×10^{-1}
Commercial and industrial [a]	3.13×10^{-1}
Boating [a]	2.28×10^{-1}
Recreation [a]	1.80×10^{-1}
**Miscellaneous offroad**	$\mathbf{3.87}\times10^{-2}$
Diesel - other	3.34×10^{-2}
Gasoline - unclassified	1.17×10^{-3}
Gasoline - public nonhighway	4.13×10^{-3}
Railroad [b]	3.64×10^{-2}
Aviation [c]	2.35×10^{-2}
Construction [a]	2.35×10^{-2}
Military [b]	1.98×10^{-2}
Agricultural [a]	1.30×10^{-3}

[a] Gasoline. [b] Distillate fuel. [c] Aviation gasoline.

S6 Additional Figures

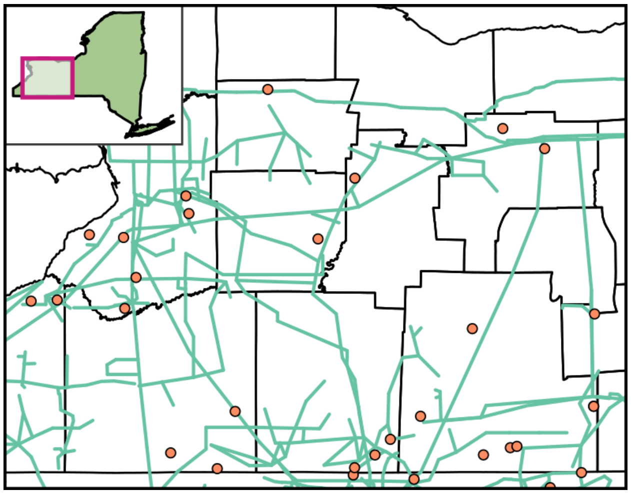

Figure S4: Locations of pipelines (green lines) and compressor stations (orange points) in New York from publicly-available datasets from the US Energy Information Administration.[93,94] Note the unrealistic distances between many of the compressor stations and the nearest pipeline in these datasets. Black lines indicate New York state and county borders. Inset of New York in the upper left indicates the area shown in the main plot.

Figure S4 shows compressor station and pipeline locations in western New York from these datasets. Compressor station coordinates are often located hundreds to thousands of meters away from any pipeline data, rendering the Energy Information Administration datasets unusable at the resolution we use for our gridded inventory.

Figure S5 shows the offset between landfill locations and gridded emissions in the EDGARv8 inventory as mentioned in Section 3.1.1 of the main text.

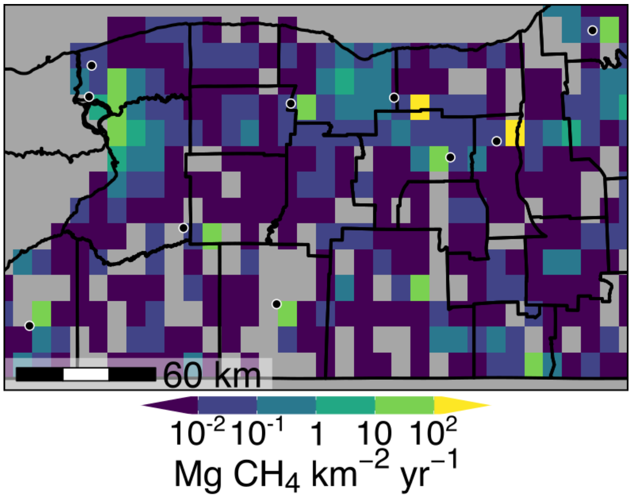

Figure S5: Landfill methane emissions from EDGARv8 compared with the locations of major landfills (black points) in upstate New York. Landfill coordinates are supplied by GHGRP[57] and confirmed with satellite imagery.[50]

S6.1 GNYS source categories at native resolution

Figures S6 through S11 show sub-regions of the 2020 NY anthropogenic methane flux gridded maps of total emissions and the five main source categories for the New York City and Rochester, NY metropolitan regions at their native 100 m horizontal resolution.

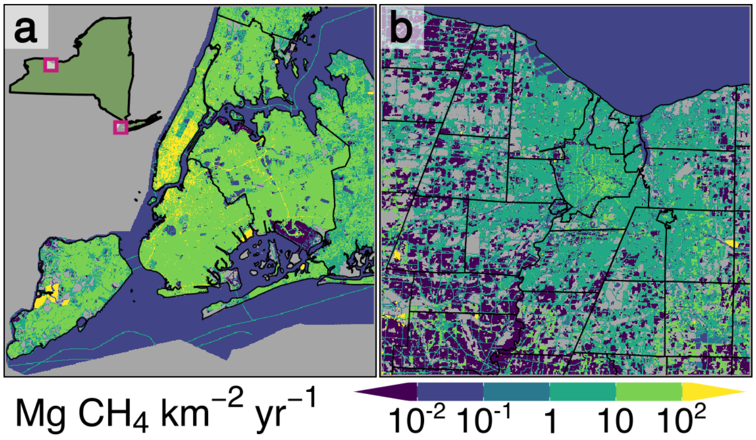

Figure S6: Spatially disaggregated 2020 total anthropogenic methane emission flux at the native resolution of 100 m×100 m for two metropolitan regions in NY: (a) New York City, and (b) Rochester. The inset showing NY State in (a) indicates the locations shown in (a) and (b) in red boxes. County/borough and city/town municipal borders are shown as black lines in each panel, respectively.

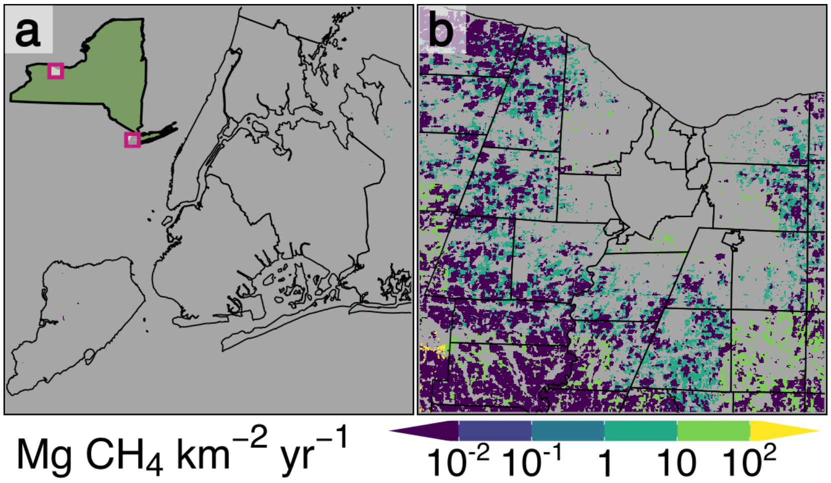

Figure S7: Spatially disaggregated 2020 New York agriculture methane emission flux at the native resolution of 100 m×100 m for two metropolitan regions in NY: (a) New York City, and (b) Rochester. The inset showing NY State in (a) indicates the locations shown in (a) and (b) in red boxes. County/borough and city/town municipal borders are shown as black lines in each panel, respectively.

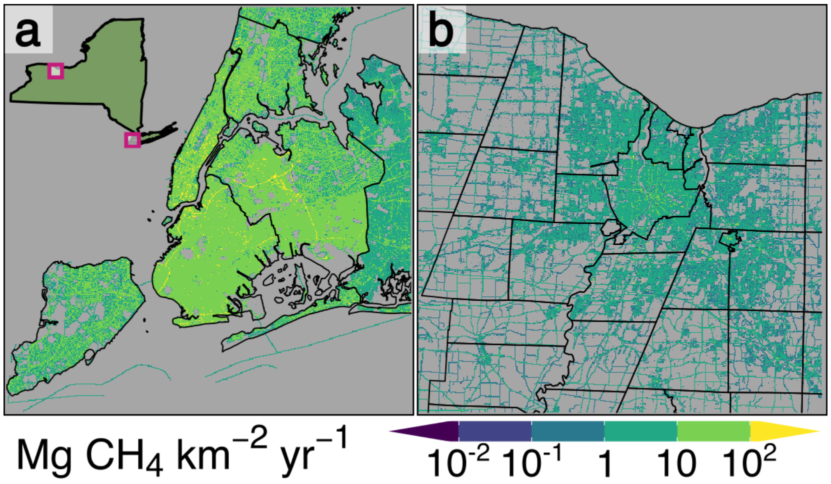

Figure S8: Spatially disaggregated 2020 New York fossil fuel systems methane emission flux at the native resolution of 100 m×100 m for two metropolitan regions in NY: (a) New York City, and (b) Rochester. The inset showing NY State in (a) indicates the locations shown in (a) and (b) in red boxes. County/borough and city/town municipal borders are shown as black lines in each panel, respectively.

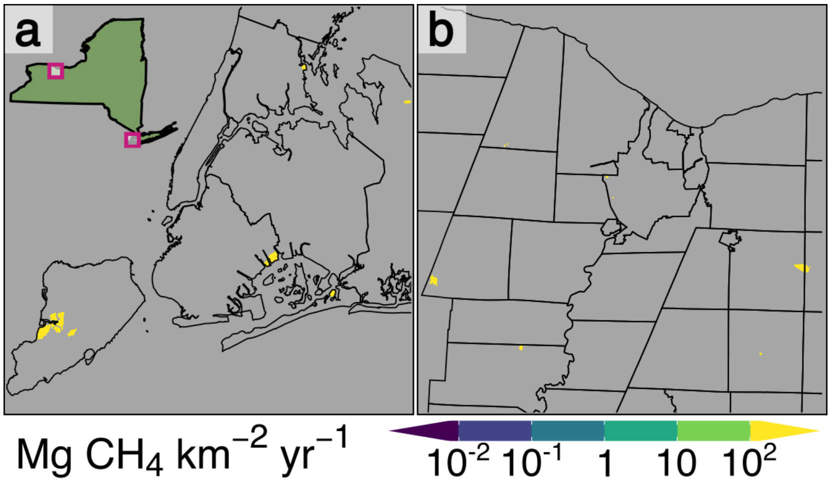

Figure S9: Spatially disaggregated 2020 New York solid waste methane emission flux at the native resolution of 100 m×100 m for two metropolitan regions in NY: (a) New York City, and (b) Rochester. The inset showing NY State in (a) indicates the locations shown in (a) and (b) in red boxes. County/borough and city/town municipal borders are shown as black lines in each panel, respectively.

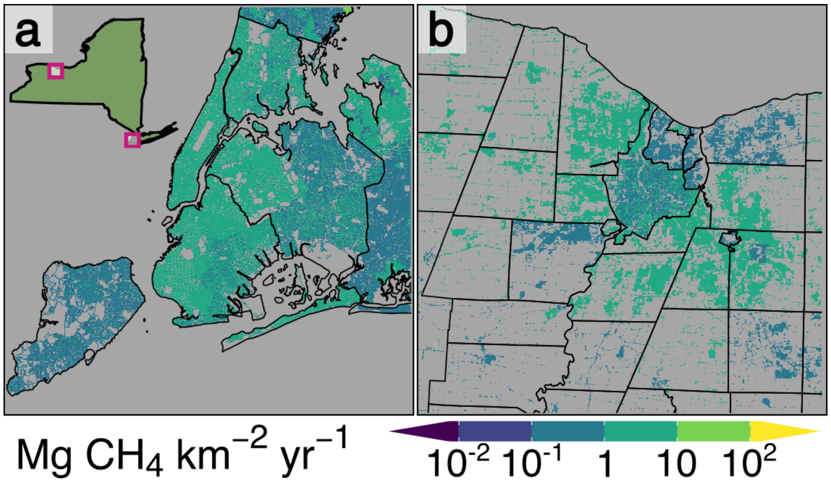

Figure S10: Spatially disaggregated 2020 New York wastewater methane emission flux at the native resolution of 100 m×100 m for two metropolitan regions in NY: (a) New York City, and (b) Rochester. The inset showing NY State in (a) indicates the locations shown in (a) and (b) in red boxes. County/borough and city/town municipal borders are shown as black lines in each panel, respectively.

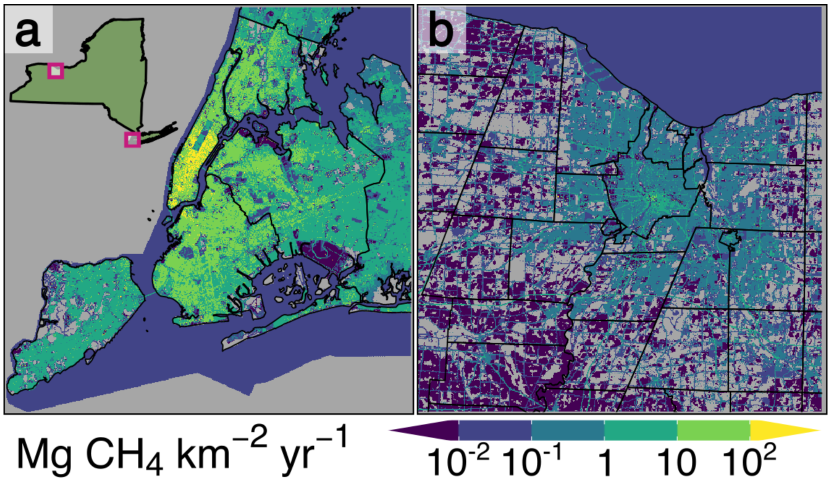

Figure S11: Spatially disaggregated 2020 New York methane emission flux from fuel combustion and industry at the native resolution of 100 m×100 m for two metropolitan regions in NY: (a) New York City, and (b) Rochester. The inset showing NY State in (a) indicates the locations shown in (a) and (b) in red boxes. County/borough and city/town municipal borders are shown as black lines in each panel, respectively.

S6.2 GEPA2 Comparisons

Figures S12 through S16 show gridded methane emission fluxes from each source category in this work compared with the 2020 Express Extension of the updated gridded EPA methane emissions inventory[4] (GEPA2). Each figure contains the following subplots: (a) emissions from the gridded New York inventories (GNYS), degraded to 0.1° horizontal resolution, (b) the GEPA2 emissions in New York only, and (c) the difference between the GNYS emissions and the GEPA2 New York emissions. Note that the color scales for subplots (a, b) are logarithmic while the color scales for subplots (c) are linear.

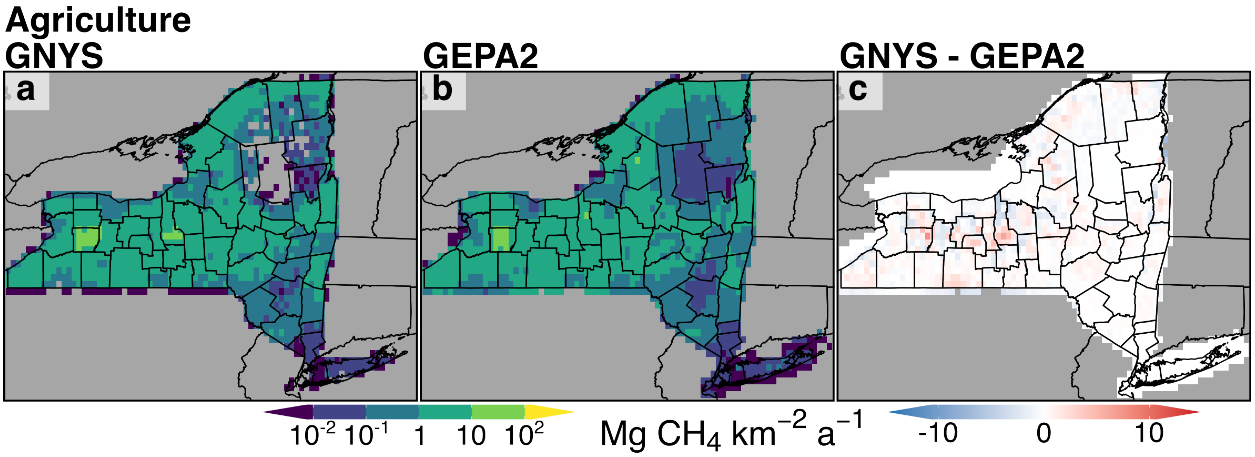

Figure S12: Anthropogenic methane emissions from agriculture

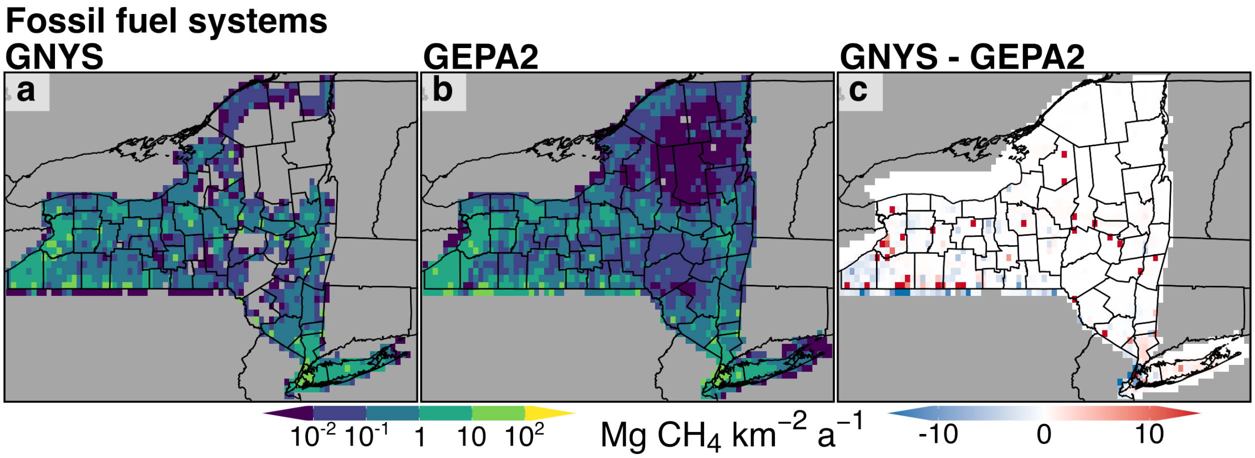

Figure S13: Anthropogenic methane emissions from fossil fuel systems

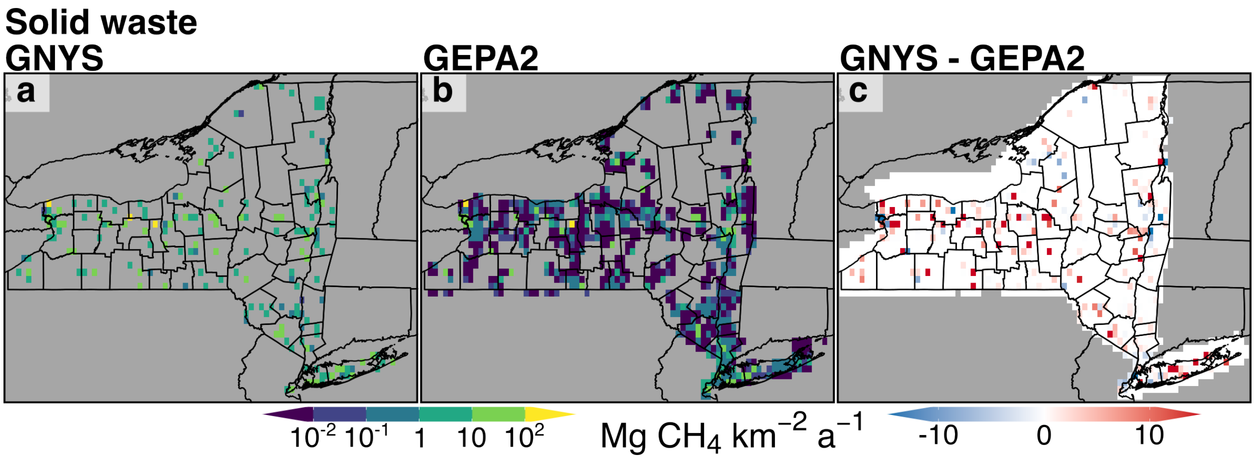

Figure S14: Anthropogenic methane emissions from solid waste

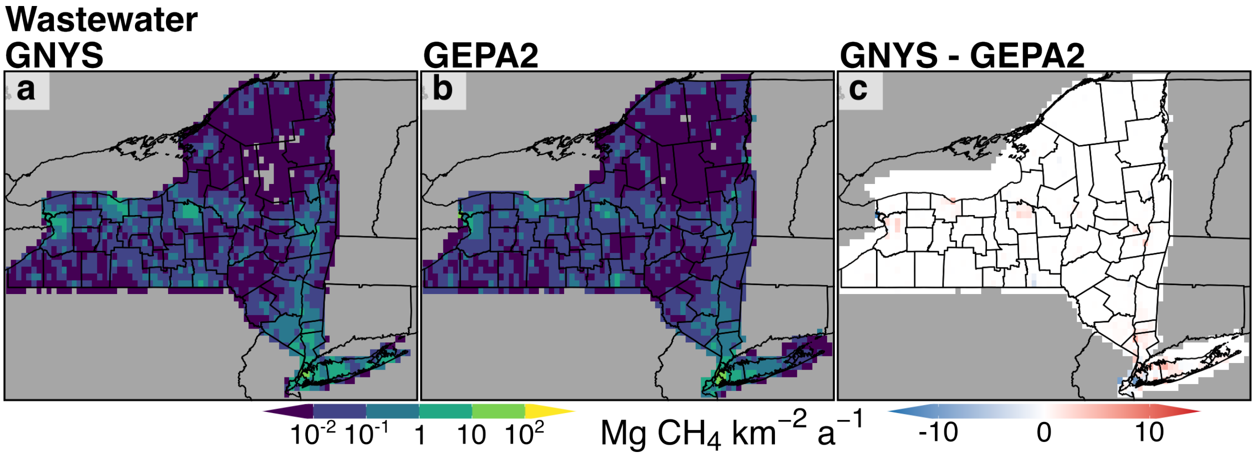

Figure S15: Anthropogenic methane emissions from wastewater

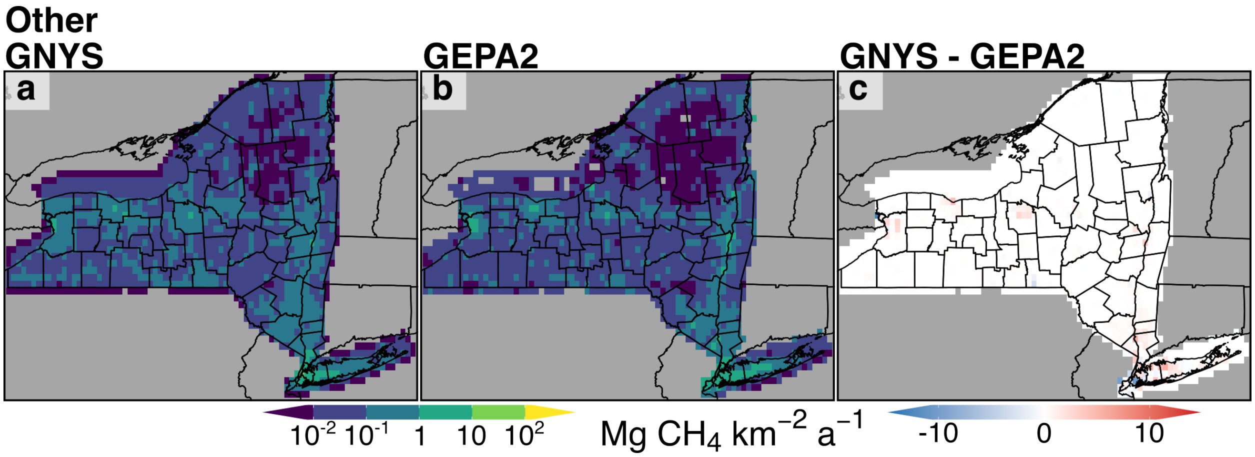

Figure S16: Anthropogenic methane emissions from fuel combustion and industry

S6.3 EDGARv8 Comparisons

The following figures show gridded methane emission fluxes from each source category in this work compared with the Emissions Database for Global Atmospheric Research version 8.0[68] (EDGARv8). Each figure contains the following subplots: (a) emissions from the gridded New York inventories (GNYS), degraded to 0.1° horizontal resolution, (b) the EDGARv8 emissions in New York only, and (c) difference between the GNYS emissions and the EDGARv8 New York emissions. Note that the color scales for subplots (a, b) are logarithmic while the color scales for subplots (c) are linear.

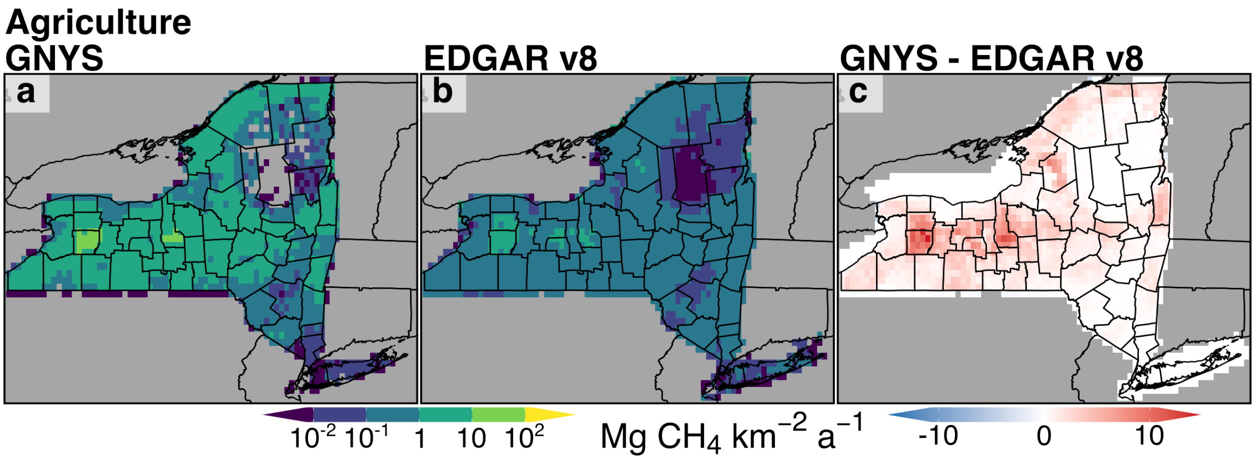

Figure S17: Anthropogenic methane emissions from agriculture

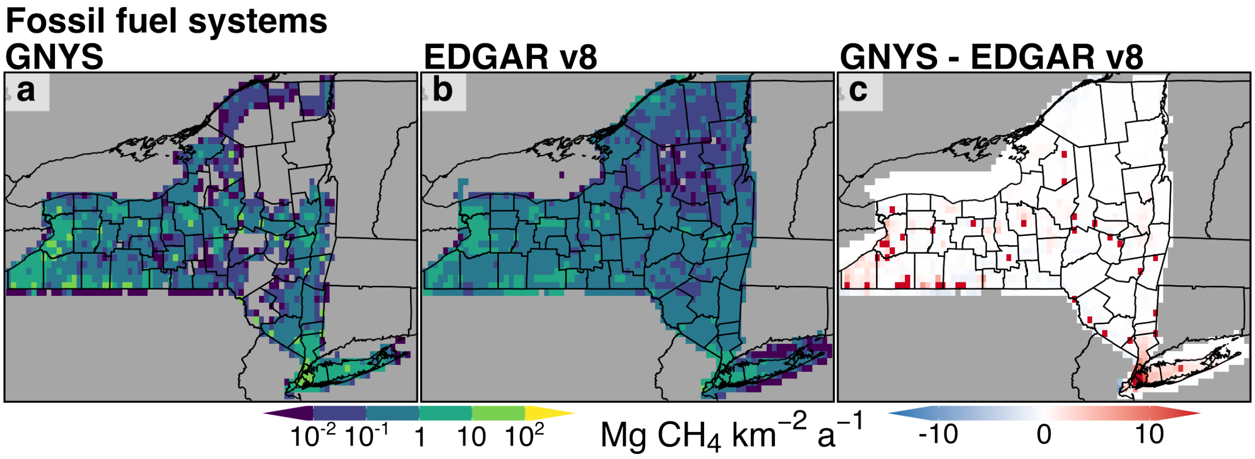

Figure S18: Anthropogenic methane emissions from fossil fuel systems

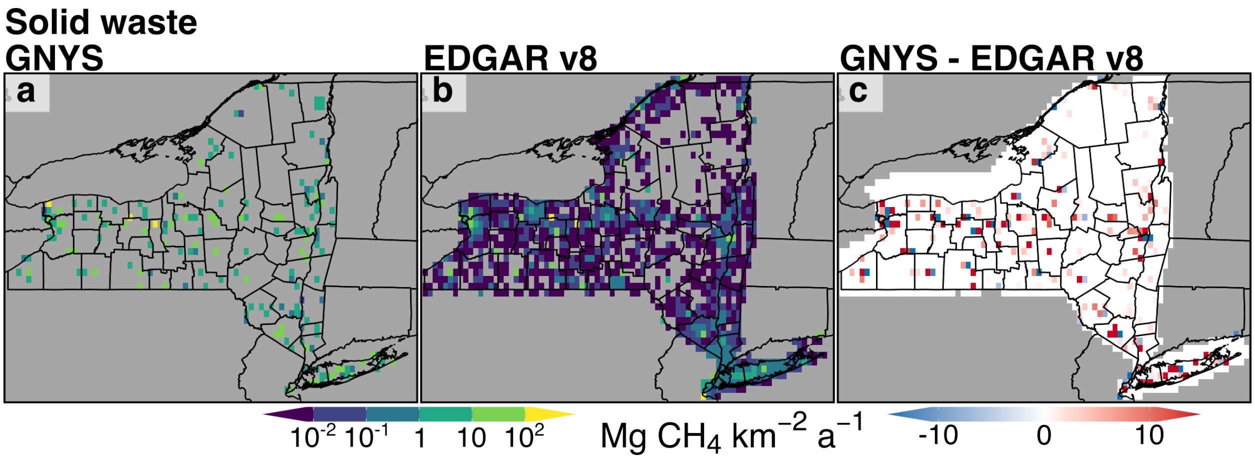

Figure S19: Anthropogenic methane emissions from solid waste

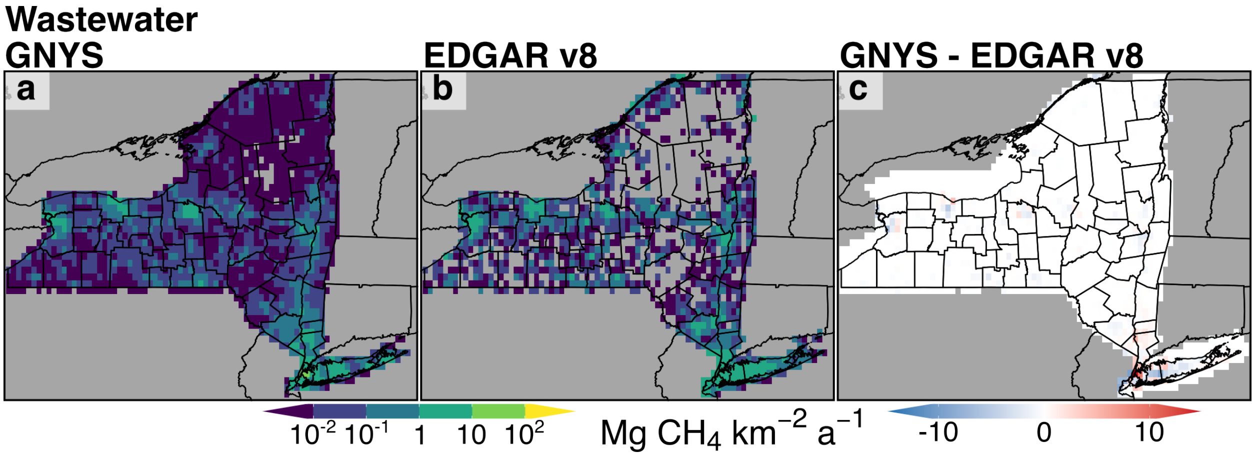

Figure S20: Anthropogenic methane emissions from wastewater

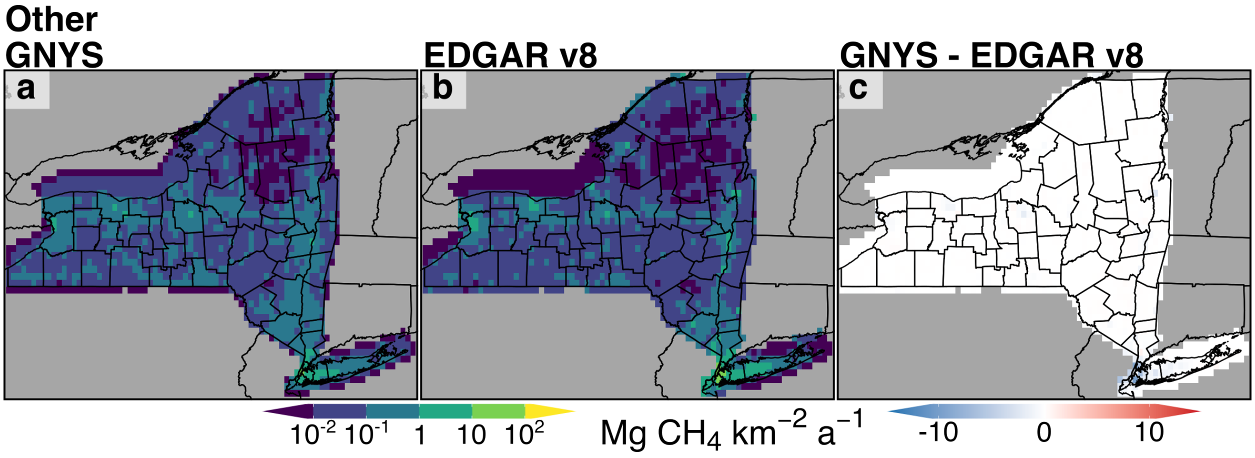

Figure S21: Anthropogenic methane emissions from fuel combustion and industry

S6.4 NY-UA Comparisons

The following figures show gridded methane emission fluxes in this work compared with the HRB gridded inventory from Pitt *et al.*[76] for each of the source categories in Table S12. Each figure contains the following subplots: (a, b) Fluxes in this work (GNYS) conservatively remapped to 0.02° horizontal resolution and gridded inventory version HRB from Pitt *et al.*[76] (NY-UA), respectively, in the overlap in their domains; (c) The difference between (a) and (b). Inset map of New York in each subplot (a) indicates the area plotted, the overlap in the domains of the NY-UA inventory and this work. Black lines in each plot show state and New York county borders. Note that the color scales for subplots (a, b) are logarithmic while the color scales for subplots (c) are linear.

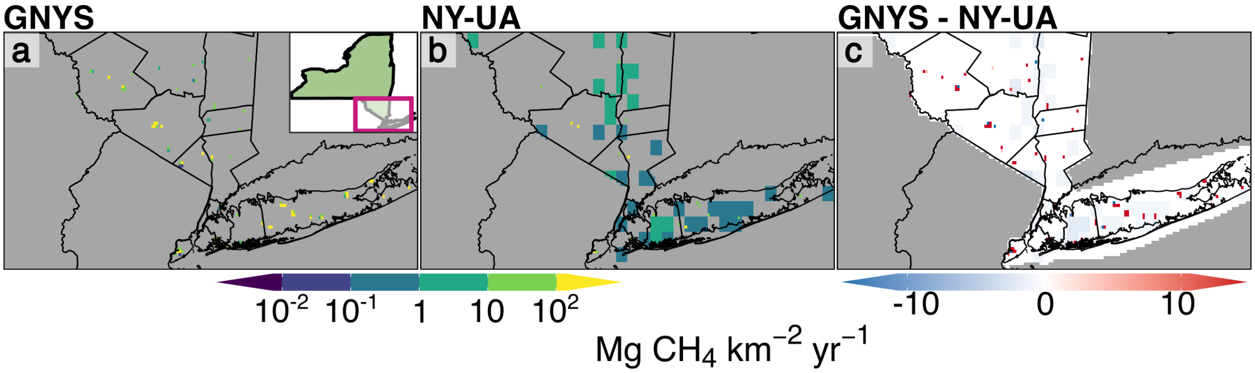

Figure S22: Comparison between anthropogenic methane emission fluxes from landfills in version HRB of the NY-UA inventory from Pitt *et al.*[76] and this work (GNYS)

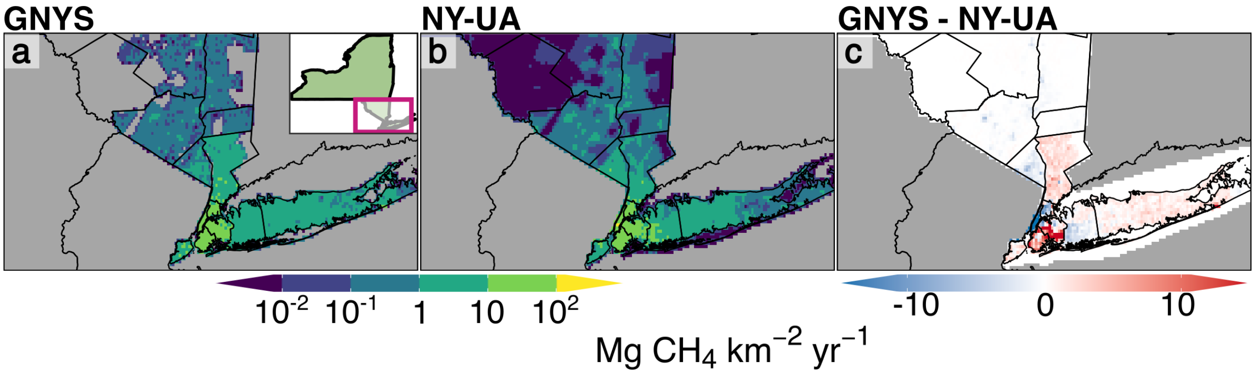

Figure S23: Comparison between anthropogenic methane emission fluxes from natural gas distribution in version HRB of the NY-UA inventory from Pitt *et al.*[76] and this work (GNYS)

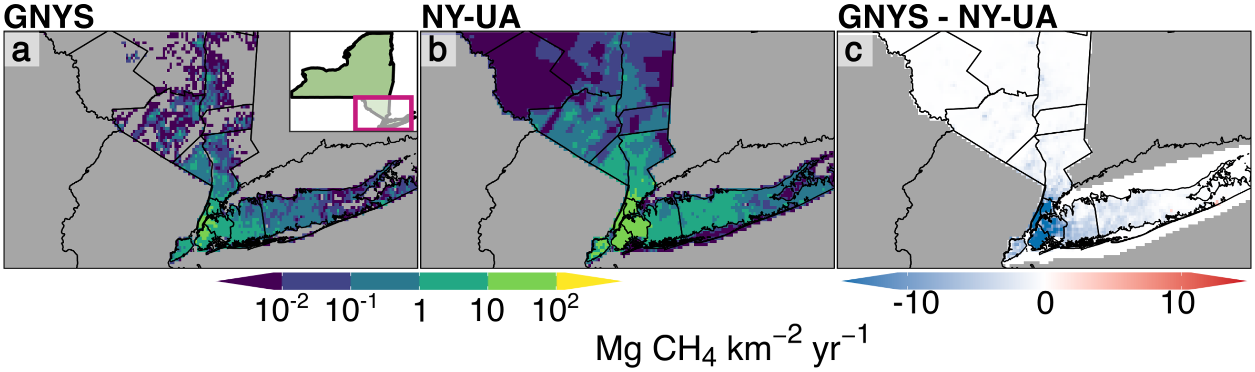

Figure S24: Comparison between anthropogenic methane emission fluxes from residential natural gas post-meter emissions in version HRB of the NY-UA inventory from Pitt *et al.*[76] and this work (GNYS)

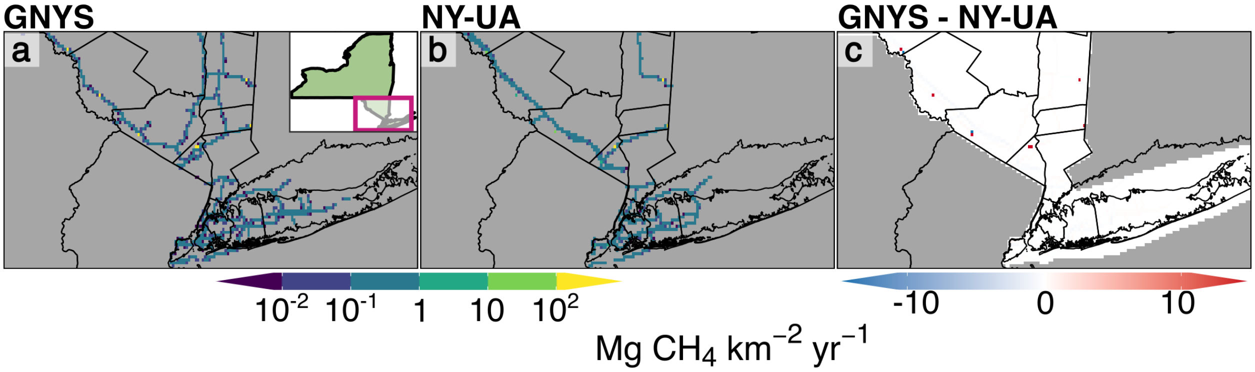

Figure S25: Comparison between anthropogenic methane emission fluxes from natural gas transmission in version HRB of the NY-UA inventory from Pitt *et al.*[76] and this work (GNYS)

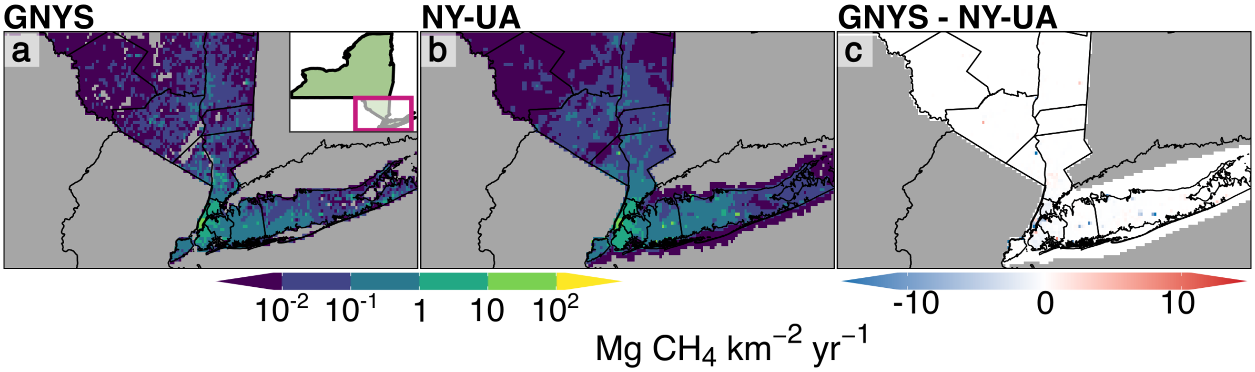

Figure S26: Comparison between anthropogenic methane emission fluxes from stationary combustion of fossil fuels in version HRB of the NY-UA inventory from Pitt *et al.*[76] and this work (GNYS)

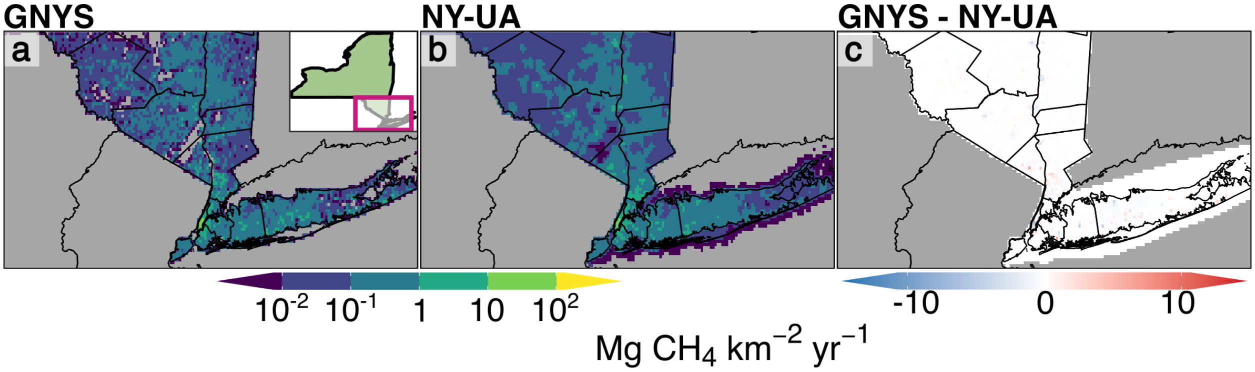

Figure S27: Comparison between anthropogenic methane emission fluxes from stationary combustion of wood in version HRB of the NY-UA inventory from Pitt *et al.*[76] and this work (GNYS)

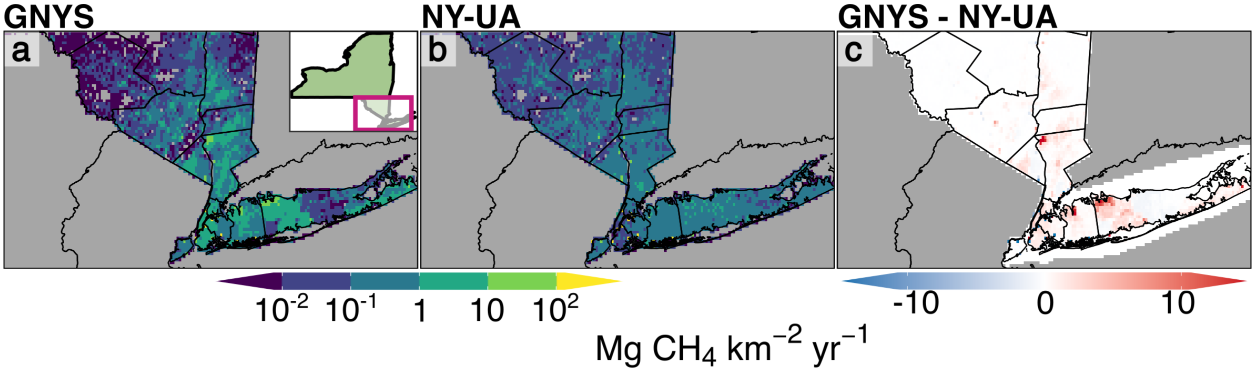

Figure S28: Comparison between anthropogenic methane emission fluxes from wastewater in version HRB of the NY-UA inventory from Pitt *et al.*[76] and this work (GNYS)

References

(1) National Geospatial-Intelligence Agency The Universal Grids and the Transverse Mercator and Polar Stereographic Map Projections NGA.SIG.0012_2.0.0_UTMUPS. 2014; https://nsgreg.nga.mil/doc/view?i=4056, (accessed 2024-09-18).

(2) USDA; NASS Census of Agriculture. 2019; https://quickstats.nass.usda.gov/, (accessed 2024-09-18).

(3) EPA Inventory of U.S. Greenhouse Gas Emissions and Sinks: 1990-2022. EPA 430R-24004. 2024; https://www.epa.gov/ghgemissions/inventory-us-greenhouse-gas-emissions-and-sinks-1990-2022, U.S. Environmental Protection Agency (accessed 2024-09-18).

(4) Maasakkers, J. D.; McDuffie, E. E.; Sulprizio, M. P.; Chen, C.; Schultz, M.; Brunelle, L.; Thrush, R.; Steller, J.; Sherry, C.; Jacob, D. J.; Jeong, S.; Irving, B.; Weitz, M. A Gridded Inventory of Annual 2012–2018 U.S. Anthropogenic Methane Emissions. *Environmental Science & Technology* **2023**, *57*, 16276–16288.

(5) Dewitz, J.; U.S. Geological Survey. National Land Cover Database (NLCD) 2019 Products (ver. 2.0, June 2021): U.S. Geological Survey data release. 2021; doi:10.5066/P9KZCM54 (accessed 2025-07-14).

(6) Yang, L.; Jin, S.; Danielson, P.; Homer, C.; Gass, L.; Bender, S. M.; Case, A.; Costello, C.; Dewitz, J.; Fry, J.; Funk, M.; Granneman, B.; Liknes, G. C.; Rigge, M.; Xian, G. A new generation of the United States National Land Cover Database: Requirements, research priorities, design, and implementation strategies. *ISPRS Journal of Photogrammetry and Remote Sensing* **2018**, *146*, 108–123.

(7) New York Natural Heritage Program New York Protected Areas Database (NYPAD). 2022; https://www.nypad.org/download, (accessed 2024-09-18).

(8) Shakoor, A.; Shakoor, S.; Rehman, A.; Ashraf, F.; Abdullah, M.; Shahzad, S. M.; Farooq, T. H.; Ashraf, M.; Manzoor, M. A.; Altaf, M. M.; Altaf, M. A. Effect of animal manure, crop type, climate zone, and soil attributes on greenhouse gas emissions from agricultural soils—A global meta-analysis. *Journal of Cleaner Production* **2021**, *278*, 124019.

(9) Ellert, B. H.; Janzen, H. H. Nitrous oxide, carbon dioxide and methane emissions from irrigated cropping systems as influenced by legumes, manure and fertilizer. *Canadian Journal of Soil Science* **2008**, *88*, 207–217.

(10) Ozlu, E.; Kumar, S. Response of surface GHG fluxes to long-term manure and inorganic fertilizer application in corn and soybean rotation. *Science of The Total Environment* **2018**, *626*, 817–825.

(11) Oladipo, D. G.; Wei, K.; Hu, L.; Medaiyese, A.; Bah, H.; Gbadegesin, L. A.; Zhu, B. Short-Term Assessment of Nitrous Oxide and Methane Emissions on a Crop Yield Basis in Response to Different Organic Amendment Types in Sichuan Basin. *Atmosphere* **2021**, *12*.

(12) Mori, A. Farmyard manure application and associated root proliferation improve the net greenhouse gas balance of Italian ryegrass – Maize double-cropping field in Nasu, Japan. *Science of The Total Environment* **2021**, *792*, 148332.

(13) Wightman, J. L.; Woodbury, P. B. *2023 New York State Agricultural Greenhouse Gas Inventory (1990-2021)*; 2024.

(14) Gavrilova, O. et al. *2019 Refinement to the 2006 IPCC Guidelines for National Greenhouse Gas Inventories*; IPCC, 2019; Vol. 4; Chapter 10.

(15) Mangino, J.; Bartram, D.; Brazy, A. Development of a methane conversion factor to estimate emissions from animal waste lagoons. Proceedings of the U.S. EPA's 17th

Annual Emission Inventory Conference, Atlanta, GA. April 15-18, 2002; United States Environmental Protection Agency (EPA).

(16) EPA Inventory of U.S. Greenhouse Gas Emissions and Sinks: 1990-2018. EPA 430-R-20-002. 2020; https://www.epa.gov/ghgemissions/inventory-us-greenhouse-gas-emissions-and-sinks-1990-2018, U.S. Environmental Protection Agency (accessed 2024-09-18).

(17) NASA Global Modeling and Assimilation Office (GMAO) MERRA-2 instM_2d_asm_Nx: 2d,Monthly mean,Single-Level,Assimilation,Single-Level Diagnostics V5.12.4. 2015; (accessed 2024-09-18).

(18) GDAL/OGR contributors GDAL/OGR Geospatial Data Abstraction software Library. Open Source Geospatial Foundation, 2024; doi:10.5281/zenodo.5884351.

(19) Gilbert, M.; Cinardi, G.; Da Re, D.; Wint, W. G. R.; Wisser, D.; Robinson, T. P. Global cattle distribution in 2015 (5 minutes of arc). 2022; https://doi.org/10.7910/DVN/LHBICE.

(20) Gilbert, M.; Cinardi, G.; Da Re, D.; Wint, W. G. R.; Wisser, D.; Robinson, T. P. Global chickens distribution in 2015 (5 minutes of arc). 2022; https://doi.org/10.7910/DVN/SXHLF3.

(21) Gilbert, M.; Cinardi, G.; Da Re, D.; Wint, W. G. R.; Wisser, D.; Robinson, T. P. Global goats distribution in 2015 (5 minutes of arc). 2022; https://doi.org/10.7910/DVN/YYG6ET.

(22) Gilbert, M.; Cinardi, G.; Da Re, D.; Wint, W. G. R.; Wisser, D.; Robinson, T. P. Global horses distribution in 2015 (5 minutes of arc). 2022; https://doi.org/10.7910/DVN/JJGCTX.

(23) Gilbert, M.; Cinardi, G.; Da Re, D.; Wint, W. G. R.; Wisser, D.; Robinson, T. P. Global pigs distribution in 2015 (5 minutes of arc). 2022; https://doi.org/10.7910/DVN/CIVCPB.

(24) Gilbert, M.; Cinardi, G.; Da Re, D.; Wint, W. G. R.; Wisser, D.; Robinson, T. P. Global sheep distribution in 2015 (5 minutes of arc). 2022; https://doi.org/10.7910/DVN/VZOYHM.

(25) New York State Department of Environmental Conservation (NYSDEC) High-Volume Hydraulic Fracturing In New York State. 2015; https://dec.ny.gov/environmental-protection/oil-gas/high-volume-hydraulic-fracturing, (accessed 2024-09-18).

(26) New York State Energy Research and Development Authority (NYSERDA) New York State Oil and Gas Sector: Methane Emissions Inventory. 2022; https://www.nyserda.ny.gov/About/Publications/Energy-Analysis-Reports-and-Studies/Greenhouse-Gas-Emissions, NYSERDA Report Number 22-38. Prepared by Abt Associates, Rockville, MD (accessed 2024-09-18).

(27) Lash, G. G.; Lash, E. P. Early history of the natural gas industry, Fredonia, New York. *Search and Discovery* **2014**, (accessed 2024-09-18).

(28) New York State Department of Environmental Conservation (NYSDEC) Drone Technology Helps Locate Orphaned Wells. https://dec.ny.gov/environmental-protection/oil-gas/orphaned-abandoned-well-plugging/drone-technology, (accessed 2024-09-18).

(29) de Smet, T. S.; Nikulin, A.; Romanzo, N.; Graber, N.; Dietrich, C.; Puliaiev, A. Successful application of drone-based aeromagnetic surveys to locate legacy oil and gas wells in Cattaraugus county, New York. *Journal of Applied Geophysics* **2021**, *186*, 104250.

(30) U.S. Census Bureau 2020 TIGER/Line Shapefiles: Roads. https://www2.census.gov/geo/tiger/TIGER2020/ROADS/, (accessed 2024-09-18).

(31) New York State Department of Public Service New York State Gas Utility Service Territories. 2023; https://data.ny.gov/Energy-Environment/NYS-Gas-Utility-Service-Territories/i7nm-ih89, (accessed 2024-09-18).

(32) US Department of Transportation - Pipeline and Hazardous Materials Safety Administration Gas Distribution Annual Data - 2010 to present. https://www.phmsa.dot.gov/data-and-statistics/pipeline/gas-distribution-gas-gathering-gas-transmission-hazardous-liquids, (accessed 2024-09-18).

(33) New York State Department of Public Service Gas Utility Companies. https://dps.ny.gov/natural-gas, (accessed 2024-09-18).

(34) New York State GIS Clearinghouse New York State Civil Boundaries. https://data.gis.ny.gov/maps/074d3456e5664f5e85d0fb251d05cc5b/about, (accessed 2024-09-18).

(35) Merrin, Z.; Francisco, P. W. Unburned Methane Emissions from Residential Natural Gas Appliances. *Environmental Science & Technology* **2019**, *53*, 5473–5482.

(36) EPA Inventory of U.S. Greenhouse Gas Emissions and Sinks: 1990-2021. EPA 430-R-23-002. 2023; https://www.epa.gov/ghgemissions/inventory-us-greenhouse-gas-emissions-and-sinks-1990-2021, U.S. Environmental Protection Agency (accessed 2024-09-18).

(37) IPCC Emission Factor Database (EFDB). https://www.ipcc-nggip.iges.or.jp/EFDB/find_ef.php, (accessed 2024-09-18).

(38) New York State ITS Geospatial Services New York State Tax Parcel Centroid Points. https://gis.ny.gov/parcels/, (accessed 2024-09-18).

(39) New York State Department of Taxation and Finance Property type classification codes. https://www.tax.ny.gov/research/property/assess/manuals/prclas.htm, (accessed 2024-09-18).

(40) New York City Department of City Planning PLUTO Data Dictionary, Appendix D: Land Use Categories. https://www.nyc.gov/assets/planning/download/pdf/data-maps/open-data/pluto_datadictionary.pdf, (accessed 2024-09-18).

(41) New York City Department of Finance NYC Building Classifications - DOF. https://www.nyc.gov/assets/finance/jump/hlpbldgcode.html, (accessed 2024-09-18).

(42) US Census Bureau *2020 Census Demographic and Housing Characteristics File (DHC)*; 2023; https://www.census.gov/data/tables/2023/dec/2020-census-dhc.html (accessed 2025-07-14).

(43) United States Energy Information Administration (EIA) Natural Gas Monthly. https://www.eia.gov/naturalgas/monthly/, (accessed 2024-09-18).

(44) New York State Department of Environmental Conservation (NYSDEC) Annual Solid Waste Management Facility Reports, 2019-2022. 2022; https://extapps.dec.ny.gov/fs/projects/SWMF/Annual%20Reports_Solid%20Waste%20Management%20Facility/, (accessed 2024-09-18).

(45) EPA Inventory of U.S. Greenhouse Gas Emissions and Sinks: 1990-2020. EPA 430-R-22-003. 2022; https://www.epa.gov/ghgemissions/inventory-us-greenhouse-gas-emissions-and-sinks-1990-2020, U.S. Environmental Protection Agency (accessed 2024-09-18).

(46) New York State Department of Environmental Conservation (NYSDEC) 2022 New York State Greenhouse Gas Emissions Report: Sectoral Report 4: Waste. 2022; https://dec.ny.gov/environmental-protection/climate-change/greenhouse-gas-emissions-report#Report, (accessed 2023-09-06).

(47) EPA State Inventory and Projection Tool - Solid Waste User's Guide. 2022; https://www.epa.gov/statelocalenergy/download-state-inventory-and-projection-tool, U.S. Environmental Protection Agency (accessed 2024-09-18).

(48) Xie, P.; Arkin, P. A. Global Precipitation: A 17-Year Monthly Analysis Based on Gauge Observations, Satellite Estimates, and Numerical Model Outputs. *Bulletin of the American Meteorological Society* **1997**, *78*, 2539–2558, CMAP Precipitation data provided by the NOAA PSL, Boulder, Colorado, USA, from their website at https://psl.noaa.gov.

(49) United States Environmental Protection Agency (EPA) Landfill Methane Outreach Program (LMOP). 2020; https://www.epa.gov/lmop, (accessed 2024-09-18).

(50) Google Google Maps satellite imagery. 2024; https://www.google.com/maps, (accessed 2024-09-18).

(51) Bartram, D.; Short, M. D.; Ebie, Y.; Farkaš, J.; Gueguen, C.; Peters, G. M.; Zanzottera, N. M.; Karthik, M. *2019 Refinement to the 2006 IPCC Guidelines for National Greenhouse Gas Inventories*; 2019; Intergovernmental Panel on Climate Change.

(52) U.S. Geological Survey National Hydrography Dataset (NHD). https://www.usgs.gov/national-hydrography/access-national-hydrography-products, (accessed 2024-09-18).

(53) Huetteman, J.; Tafoya, J.; Johnson, T.; Schreifels, J. EPA-EIA Power Sector Data Cross-

walk. 2021; https://www.epa.gov/airmarkets/power-sector-data-crosswalk, (accessed 2024-09-18).

(54) United States Environmental Protection Agency (EPA) Clean Air Markets Program Data. https://campd.epa.gov/, Washington, DC: Office of Atmospheric Protection, Clean Air Markets Division (accessed 2024-09-18).

(55) United States Energy Information Administration (EIA) Form EIA-860 Detailed Data. https://www.eia.gov/electricity/data/eia860/, (accessed 2024-09-18).

(56) United States Energy Information Administration (EIA) Form EIA-923 Detailed Data. https://www.eia.gov/electricity/data/eia923/, (accessed 2024-09-18).

(57) US EPA Greenhouse Gas Reporting Program (GHGRP). 2020; https://www.epa.gov/ghgreporting, (accessed 2024-09-18).

(58) U.S. Department of Transportation Federal Highway Administration Highway Statistics 2020 5.4.1. Vehicle-miles of travel, by functional system. 2022; https://www.fhwa.dot.gov/policyinformation/statistics/2020/, (accessed 2024-09-18).

(59) Wightman, J. L.; Woodbury, P. B. New York Dairy Manure Management Greenhouse Gas Emissions and Mitigation Costs (1992–2022). *Journal of Environmental Quality* **2016**, *45*, 266–275.

(60) McKain, K.; Down, A.; Raciti, S. M.; Budney, J.; Hutyra, L. R.; Floerchinger, C.; Herndon, S. C.; Nehrkorn, T.; Zahniser, M. S.; Jackson, R. B.; Phillips, N.; Wofsy, S. C. Methane emissions from natural gas infrastructure and use in the urban region of Boston, Massachusetts. *Proceedings of the National Academy of Sciences* **2015**, *112*, 1941–1946.

(61) Ren, X. et al. Methane Emissions From the Baltimore-Washington Area Based on

Airborne Observations: Comparison to Emissions Inventories. *Journal of Geophysical Research: Atmospheres* **2018**, *123*, 8869–8882.

(62) Duren, R. M. et al. California's methane super-emitters. *Nature* **2019**, *575*, 180–184.

(63) Nesser, H.; Jacob, D. J.; Maasakkers, J. D.; Lorente, A.; Chen, Z.; Lu, X.; Shen, L.; Qu, Z.; Sulprizio, M. P.; Winter, M.; Ma, S.; Bloom, A. A.; Worden, J. R.; Stavins, R. N.; Randles, C. A. High-resolution US methane emissions inferred from an inversion of 2019 TROPOMI satellite data: contributions from individual states, urban areas, and landfills. *Atmospheric Chemistry and Physics* **2024**, *24*, 5069–5091.

(64) Dogniaux, M.; Maasakkers, J. D.; Girard, M.; Jervis, D.; McKeever, J.; Schuit, B. J.; Sharma, S.; Lopez-Noreña, A.; Varon, D. J.; Aben, I. 2024, DOI: doi:10.31223/X5TB09, Preprint on EarthArXiv (accessed 2024-09-18).

(65) Cusworth, D. H. et al. Quantifying methane emissions from United States landfills. *Science* **2024**, *383*, 1499–1504.

(66) New York State Governor's Office Governor Cuomo Signs the 'New York State on PAUSE' Executive Order. https://www.governor.ny.gov/news/governor-cuomo-signs-new-york-state-pause-executive-order, (accessed 2024-09-18).

(67) US EPA Clean Watersheds Needs Survey. 2012; https://www.epa.gov/cwns/clean-watersheds-needs-survey-cwns-2012-report-and-data, (accessed 2024-09-18).

(68) European Commission et al. *GHG emissions of all world countries – 2023*; Publications Office of the European Union, 2023.

(69) Schulzweida, U. CDO User Guide. 2023; Zenodo. doi:10.5281/zenodo.10020800 (accessed 2025-07-23).

(70) Janssens-Maenhout, G. et al. EDGAR v4.3.2 Global Atlas of the three major greenhouse gas emissions for the period 1970–2012. *Earth System Science Data* **2019**, *11*, 959–1002.

(71) Joint Research Centre; Institute for Environment and Sustainability; Pagliari, V.; Guizzardi, D.; Janssens-Maenhout, G.; Muntean, M. *Global emission inventories in the Emission Database for Global Atmospheric Research (EDGAR) – Manual (I). I., Gridding: EDGAR emissions distribution on global gridmaps*; Publications Office of the European Union, 2012.

(72) US Department of Transportation - Bureau of Transportation Statistics Navigable Waterway Network Lines. https://data-usdot.opendata.arcgis.com/datasets/usdot::navigable-waterway-network-lines/, (accessed 2024-09-18).

(73) Zimmerle, D. J.; Williams, L. L.; Vaughn, T. L.; Quinn, C.; Subramanian, R.; Duggan, G. P.; Willson, B.; Opsomer, J. D.; Marchese, A. J.; Martinez, D. M.; Robinson, A. L. Methane Emissions from the Natural Gas Transmission and Storage System in the United States. *Environmental Science & Technology* **2015**, *49*, 9374–9383.

(74) Hart Energy Rextag Natural Gas Dataset, 2022-08 to 2023-08. 2023; https://rextag.com/natural-gas-amp-misc, (accessed 2023-07-31).

(75) Ravikumar, A.; Li, Z. H.; Yang, S. L.; Smith, M. 2024, DOI: doi:10.26434/chemrxiv-2024-8jmtn, Preprint on ChemRxiv (accessed 2024-09-18).

(76) Pitt, J. R. et al. Underestimation of Thermogenic Methane Emissions in New York City. *Environmental Science & Technology* **2024**, *58*, 9147–9157.

(77) Gately, C.; Hutyra, L. Anthropogenic Carbon Emission System, 2012-2017, Version 2. 2022; doi:10.3334/ORNLDAAC/1943 (accessed 2025-07-23).

(78) Gurney, K.; Liang, J.; Patarasuk, R.; Song, Y.; Huang, J.; Roest, G. Vulcan: High-

Resolution Annual Fossil Fuel CO2 Emissions in USA, 2010-2015, Version 3. 2020; https://daac.ornl.gov/cgi-bin/dsviewer.pl?ds_id=1741.

(79) Bloom, A.; Bowman, K.; Lee, M.; Turner, A.; Schroeder, R.; Worden, J.; Weidner, R.; McDonald, K.; Jacob, D. CMS: Global 0.5-deg Wetland Methane Emissions and Uncertainty (WetCHARTs v1.3.1). 2021; https://daac.ornl.gov/cgi-bin/dsviewer.pl?ds_id=1915.

(80) Bridgham, S. D.; Megonigal, J. P.; Keller, J. K.; Bliss, N. B.; Trettin, C. In *The First State of the Carbon Cycle Report (SOCCR): The North American Carbon Budget and Implications for the Global Carbon Cycle*; King, A. W., Dilling, L., Zimmerman, G. P., Fairman, D. M., Houghton, R. A., Marland, G., Rose, A. Z., , Wilbanks, T. J., Eds.; National Oceanic and Atmospheric Administration, National Climatic Data Center, Asheville, NC, USA, 2007; pp 177–192.

(81) U.S. Fish and Wildlife Service National Wetlands Inventory. https://www.fws.gov/program/national-wetlands-inventory/data-download, (accessed 2024-09-18).

(82) Weller, Z. D.; Hamburg, S. P.; von Fischer, J. C. A National Estimate of Methane Leakage from Pipeline Mains in Natural Gas Local Distribution Systems. *Environmental Science & Technology* **2020**, *54*, 8958–8967.

(83) Lamb, B. K.; Edburg, S. L.; Ferrara, T. W.; Howard, T.; Harrison, M. R.; Kolb, C. E.; Townsend-Small, A.; Dyck, W.; Possolo, A.; Whetstone, J. R. Direct Measurements Show Decreasing Methane Emissions from Natural Gas Local Distribution Systems in the United States. *Environmental Science & Technology* **2015**, *49*, 5161–5169.

(84) United States Environmental Protection Agency (EPA) 2017 National Emissions Inventory (NEI) Data EPA-454/R-21-001. 2021; https://www.epa.gov/air-emissions-inventories/2017-national-emissions-inventory-nei-data, (accessed 2024-09-18).

(85) Hajny, K. D.; Salmon, O. E.; Rudek, J.; Lyon, D. R.; Stuff, A. A.; Stirm, B. H.; Kaeser, R.; Floerchinger, C. R.; Conley, S.; Smith, M. L.; Shepson, P. B. Observations of Methane Emissions from Natural Gas-Fired Power Plants. *Environmental Science & Technology* **2019**, *53*, 8976–8984, PMID: 31283190.

(86) Plant, G.; Kort, E. A.; Floerchinger, C.; Gvakharia, A.; Vimont, I.; Sweeney, C. Large Fugitive Methane Emissions From Urban Centers Along the U.S. East Coast. *Geophysical Research Letters* **2019**, *46*, 8500–8507.

(87) Floerchinger, C.; Shepson, P. B.; Hajny, K.; Daube, B. C.; Stirm, B. H.; Sweeney, C.; Wofsy, S. C. Relative flux measurements of biogenic and natural gas-derived methane for seven U.S. cities. *Elementa: Science of the Anthropocene* **2021**, *9*, 000119.

(88) Sargent, M. R.; Floerchinger, C.; McKain, K.; Budney, J.; Gottlieb, E. W.; Hutyra, L. R.; Rudek, J.; Wofsy, S. C. Majority of US urban natural gas emissions unaccounted for in inventories. *Proceedings of the National Academy of Sciences* **2021**, *118*, e2105804118.

(89) New York State Department of Environmental Conservation (NYSDEC) 2022 New York State Greenhouse Gas Emissions Report. 2022; https://dec.ny.gov/environmental-protection/climate-change/greenhouse-gas-emissions-report#Report, (accessed 2023-09-06).

(90) New York State Energy Research and Development Authority (NYSERDA) Energy Sector Greenhouse Gas Emissions under the New York State Climate Act: 1990-2020 Report. 2022; https://www.nyserda.ny.gov/About/Publications/Energy-Analysis-Reports-and-Studies/Greenhouse-Gas-Emissions, NYSERDA Report Number 23-02. Prepared by Eastern Research Group, Inc, Concord, MA (accessed 2024-09-18).

(91) New York State Department of Environmental Conservation (NYSDEC) 2022 New York State Greenhouse Gas Emissions Report: Sectoral Report 3: Agriculture,

Forestry, and Land Use. 2022; https://dec.ny.gov/environmental-protection/climate-change/greenhouse-gas-emissions-report#Report, (accessed 2023-09-06).

(92) New York State Department of Environmental Conservation (NYSDEC) 2022 New York State Greenhouse Gas Emissions Report: Sectoral Report 2: Industrial Processes and Product Use. 2022; https://dec.ny.gov/environmental-protection/climate-change/greenhouse-gas-emissions-report, (accessed 2023-09-06).

(93) Energy Information Administration (EIA) Natural Gas Pipelines. https://atlas.eia.gov/datasets/eia::natural-gas-interstate-and-intrastate-pipelines/about, (accessed 2024-09-18).

(94) Energy Information Administration (EIA), Homeland Infrastructure Foundation-Level Data (HIFLD) Natural Gas Compressor Stations. https://hifld-geoplatform.hub.arcgis.com/datasets/geoplatform::natural-gas-compressor-stations/about, (accessed 2024-05-30).
